# DNp73 enhances tumor progression and immune evasion in multiple myeloma by targeting the MYC and MYCN pathways

**DOI:** 10.3389/fimmu.2024.1470328

**Published:** 2024-09-24

**Authors:** Lanting Liu, Dasen Gong, Hao Sun, Fangshuo Feng, Jie Xu, Xiyue Sun, Lixin Gong, Zhen Yu, Teng Fang, Yan Xu, Rui Lyu, Tingyu Wang, Wentian Wang, Wenzhi Tian, Lugui Qiu, Gang An, Mu Hao

**Affiliations:** ^1^ State Key Laboratory of Experimental Hematology, National Clinical Research Center for Blood Diseases, Haihe Laboratory of Cell Ecosystem, Institute of Hematology & Blood Diseases Hospital, Chinese Academy of Medical Sciences & Peking Union Medical College, Tianjin, China; ^2^ Tianjin Institutes of Health Science, Tianjin, China; ^3^ Department of Neurosurgery, Tianjin Medical University General Hospital, Tianjin, China; ^4^ Tianjin Neurological Institute, Tianjin, China; ^5^ ImmuneOnco Biopharmaceuticals (Shanghai) Inc., Shanghai, China; ^6^ Gobroad Healthcare Group, Beijing, China

**Keywords:** immune evasion, plasma cell malignancy, tumor progression, DNp73, MYCN, MYC

## Abstract

**Introduction:**

Multiple myeloma (MM) is an incurable hematological malignancy with high chromosome instability and heavy dependence on the immunosuppressive bone marrow microenvironment. P53 mutations are adverse prognostic factors in MM; however, clinically, some patients without P53 mutations also exhibit aggressive disease progression. DNp73, an inhibitor of TP53 tumor suppressor family members, drives drug resistance and cancer progression in several solid malignancies. Nevertheless, the biological functions of DNp73 and the molecular mechanisms in myelomagenesis remain unclear.

**Methods:**

The effects of DNp73 on proliferation and drug sensitivity were assessed using flow cytometry and xenograft models. To investigate the mechanisms of drug resistance, RNA-seq and ChIP-seq analyses were performed in MM cell lines, with validation by Western blot and RT-qPCR. Immunofluorescence and transwell assays were used to assess DNA damage and cell invasion in MM cells. Additionally, in vitro phagocytosis assays were conducted to confirm the role of DNp73 in immune evasion.

**Results:**

Our study found that activation of NF-κB-p65 in multiple myeloma cells with different p53 mutation statuses upregulates DNp73 expression at the transcriptional level. Forced expression of DNp73 promoted aggressive proliferation and multidrug resistance in MM cells. Bulk RNA-seq analysis was conducted to assess the levels of MYCN, MYC, and CDK7. A ChIP-qPCR assay was used to reveal that DNp73 acts as a transcription factor regulating MYCN gene expression. Bulk RNA-seq analysis demonstrated increased levels of MYCN, MYC, and CDK7 with forced DNp73 expression in MM cells. A ChIP-qPCR assay revealed that DNp73 upregulates MYCN gene expression as a transcription factor. Additionally, DNp73 promoted immune evasion of MM cells by upregulating MYC target genes CD47 and PD-L1. Blockade of the CD47/SIRPα and PD-1/PD-L1 signaling pathways by the SIRPα-Fc fusion protein IMM01 and monoclonal antibody atezolizumab significantly restored the anti-MM activity of macrophages and T cells in the microenvironment, respectively.

**Discussion:**

In summary, our study demonstrated for the first time that the p53 family member DNp73 remarkably induces proliferation, drug resistance, and immune escape of myeloma cells by directly targeting MYCN and regulating the MYC pathway. The oncogenic function of DNp73 is independent of p53 status in MM cells. These data contribute to a better understanding of the function of TP53 and its family members in tumorigenesis. Moreover, our study clarified that DNp73 overexpression not only promotes aggressive growth of tumor cells but, more importantly, promotes immune escape of MM cells through upregulation of immune checkpoints. DNp73 could serve as a biomarker for immunotherapy targeting PD-L1 and CD47 blockade in MM patients.

## Introduction

Multiple myeloma (MM) is a clonal plasma cell disorder. The incorporation of novel agents into upfront therapy and the introduction of maintenance approaches have improved the outcome of patients with MM. Unfortunately, MM remains incurable, and relapse is common (1, 2). The BM microenvironment is thought to play a central role in the development and progression of MM. There is an extensive crosstalk between the BM non-tumor cells and MM cells that promote myeloma cell survival (3). Role of immune checkpoints, especially PD-L1 and CD47 are important for immune escape of cancers including MM. In the last years, studies and research have been concentrating on creating new drugs recognized as Immune Checkpoint Inhibitors (ICIs) that have been launched in clinical practice to treat patients with several types of cancer, including myeloma. However, the outcome of ICI treatment is unsatisfied in clinic course (4–7).

TP73 is a member gene of the TP53 family. Unlike TP53, TP73 present duel opposite roles in the pathogenesis of cancer. Transcriptionally active p73 isoforms (TAp73) acts as tumor suppressor gene, but the dominant-negative p73 isoforms (DNp73) works as oncogene in many types of solid tumor (8). Loss of p53 function through mutations in p53 itself is a common feature in most human cancers. Many studies support that the mutant p53 proteins acquire oncogenic properties conversely that enable them to promote invasion, metastasis, proliferation and cell survival (9). Except TP53, the other family member, including TP63, TP73 also play pivotal roles in caner (10). But for p73, the mutation is extremely rare in cancers. The proapoptotic activities of TAp73 are commonly inhibited by the increased expression of DNp73 isoforms. Notably, TAp73 can partially compensate for the loss of p53 function in cancer (11). DNp73 inhibits p53 and TAp73 activities and drives drug resistance and cancer progression have been reported in several solid malignancies (12). Although DNp73 lacks a classical amino-terminal transactivation (TA) domain, it has been shown to retain TA potential due to another putative TA domain (13–16). However, the role of DNp73 in MM pathogenesis and whether it correlates with TP53 action have not been fully understood.

Our previous study uncovered that miRNA-15a is a tumor suppressor microRNA involved in the aggressive proliferation and drug resistance of MM cells. Downregulation of miRNA-15a is significantly associated with disease progression and inferior outcomes in patients with MM (17, 18). miR-15a/16-1 deletion in activated B cells promotes plasma cell and mature B-cell neoplasms (19). Another study revealed that miR-15a/16-1 regulates the proliferation of MM cells by suppressing the activity of the NF-κB activator MAP3KIP3 (20). RelA (p65) antagonizes p53 activation through sequestration of the p300 and CREB-binding protein coactivators (21). In this study, we found that the overexpression of DNp73 promotes the invasive proliferation and multidrug resistance of MM cells. Downregulation of miRNA-15a in MM cells activates NF-κB-p65, leading to increased expression of DNp73 at the transcriptional level. DNp73 is identified as a potential oncogene involved in the pathogenesis of MM, promoting disease progression and immune evasion through targeting the MYC and MYCN pathways. The results demonstrated in this study will help further refine the treatment strategy of immune checkpoint inhibitors in hematological and solid malignancies.

## Materials and methods

### Patient samples

Bone marrow samples were obtained from newly diagnosed multiple myeloma (NDMM, n=15) and relapsed/refractory multiple myeloma (RRMM, n=7) patients at the Institute of Hematology and Blood Disease Hospital (Tianjin, China). Written informed consent was obtained from all participants prior to sample collection. **Supplementary Table 1** shows the detailed characteristics of the patients. Plasma cells were isolated from the mononuclear cells of BM samples using CD138 MicroBeads (Miltenyi Biotec).

### Cell lines

Human MM cell lines (RPMI-8226, OPM2, U266, MM.1S, KMS11, NCI-H929), leukemia cell lines (K562) and non-Hodgkin’s B-cell line (DoHH2) were purchased from American Type Culture Collection. Other cell lines HEK293T, RPMI 8226/Dox40, ANBL6, OCI-MY5, ARD and ARP1 were obtained from State Key Laboratory of Experimental Hematology (2, 22). All tumor cell lines were maintained in RPMI 1640 medium (Invitrogen). All media were supplemented with 10% fetal bovine serum (Invitrogen) and 1x P/S. HEK293T cells were maintained in DMEM medium (Invitrogen) supplemented with 10% FBS and 1x P/S.

### Myeloma xenografted models

NOD/SCID female mice (5-6 weeks old) were purchased from Beijing Vital River Laboratory Animal Technology Co., Ltd. They were housed and experiments were conducted under the approval of the Animal Experiment Ethics Committee of the Institute of Hematology and Blood Diseases Hospital. *In vivo* animal experiments were performed based on our previous studies (2, 18, 23). NOD/SCID mice were subcutaneously inoculated with 1.0×10^6^ MM cells in 100 μL phosphate buffered saline (PBS) at the abdomen site for tumor induction. For the DNp73-OE group, EV and DNp73-OE ARP1 cells were used for inoculation in mice. Similarly, for the DNp73-sh group, scramble and sh-DNp73 RPMI 8226/Dox40 cells were utilized for mouse inoculation. After a period of 10 days post-inoculation, mice were randomly divided into either four groups or eight groups, consisting of four or five mice per group respectively. Subsequently, these groups received treatment with Epirubicin (3mg/kg), Pomalidomide (5mg/kg), Carfilzomib (1mg/kg) or PBS twice a week as appropriate to their respective experimental conditions. Tumor volume was measured every seven days for DNp73-OE group and every ten days for sh-DNp73 group using the formula: tumor volume = 1/2 × length ×width^2^ to assess tumor burden accurately over time. Mice were euthanized when their tumors reached a volume close to approximately 2000 mm^3^>

### Phagocytosis assay

Macrophages were derived from bone marrow mononuclear cells of MM patients. BMNC was enriched by adherence to plates for 1h at 37°C in FBS free RPMI 1640 medium. Then, non-adherent cells were removed by extensive washing with PBS. Monocytes were cultured in complete RPMI 1640 medium containing10ng/ml recombinant human M-CSF (Pepro Tech) to induce macrophages. Human macrophages were harvested for phagocytosis assay on day 7 (24). Myeloma Cells were CFSE (ThermoFisher) labeled according to the manufacturer’s instruction and incubated with human macrophages in the presence of CD47 antibody or control antibody for 2h at 37°C. Cells were washed with serum-free media repeatedly and resuspended in 200μl media. Phagocytic index was determined by counting the number of phagocytosed tumor cells per individual macrophages using the High-content screening system (PerkinElmer). The phagocytic index was calculated by the equation: Phagocytic index = (number of engulfed myeloma cells/number of macrophages) × 100. The number of macrophages analyzed more than 3000 per well.

### Western blot analysis

Western blot analyses were performed as described previously (25). Whole-cell lysates extracted from various control and experimental samples were subjected to Western blot analysis, followed by protein separation using SDS-PAGE and transfer onto PVDF membranes. The membranes were blocked with 5% non-fat milk in TRIS-buffered saline with 0.05% Tween-20 (TBST) and then incubated with primary antibodies overnight at 4°C. The appropriate HRP-conjugated secondary antibodies were added, and protein signals were detected using enhanced chemiluminescence reagents (Thermo-Scientific). The developed images were analyzed using the ChemiDoc™ software from Bio-Rad, USA. The primary antibodies were listed in **Supplementary Table 2**.

### Plasmids and transfection

The PMIRH15a-PA-1 vector was purchased from System Biosciences. DNp73 shRNA was purchased from Beijing Genomics Institute. shRNA1 sequences: 5′- CCCGCTCTTGAAGAAACTCTA -3′; shRNA2 sequences: 5′- CCAAGGGTTACAGAGCATTTA -3′. The non-sense sequencing was utilized as scramble control. shRNA sequences for DNp73 were inserted into EcoRI and AgeI sites of the doxycycline-inducible vector PLKO-Tet-on. The plasmid pCDH-CMV-DNp73-EF1-copGFP of DNp73-OE was generously provided by Dr. Carlos Pipaón (Hospital Universitario Marqués de Valdecilla-IDIVAL, Santander, Spain). Virus was prepared with the packaging system in HEK293T cells and used for the infection of cells, following by drug selection as previously described.

### Reagents

The following cytotoxic agents were used in this study: doxycycline, Bortezomib, Carfilzomib, Lenalidomide, Epirubicin, Melphalan and Bendamustine that were purchased from Selleck Chemicals, Houston, TX, USA. Atezolizumab (HY-P9904) was purchased from MedChemExpress. Drugs were dissolved in dimethylsulfoxide and subsequently diluted in cell culture medium (10% fetal bovine serum) immediately before use. The maximum final concentration of dimethylsulfoxide (<0.1%) did not affect cell proliferation and did not induce cytotoxicity on the cell lines tested. Dynabeads Human T-Activator CD3/CD28 (cat#11131D, Gibco). IMM01 was provided by Dr. Wenzhi Tian, ImmuneOnco Biopharmaceuticals (Shanghai) Inc.

### Cell proliferation assay

Stably or transiently transfected cells were used for cell growth assays. The proliferation assay was performed in accordance with the manufacturer’s instructions (CCK8-Kit; Dojindo).

### Colony-formation assays

Cells were infected with lentivirus or treated with the indicated compound for a designated time. Cells (5,000) were seeded in 24-well plates and counted 2 weeks later. Colonies containing more than 50 cells were counted.

### Response to radiation

The ARP1 and RPMI 8226/Dox40 cell lines were fed fresh medium 1 day before the experiment. The following day cells were harvested and counted. Cells were exposed to γ-radiation using a 137Cs source at a dose of 2 Gy at a dose rate of 1.209 Gy/min. After irradiation the cells were further incubated at 37°C for 8 hr. At the end of the incubation, cells were harvested and resuspended in 1 ml medium. To measure the viability of the cells the dyed trypan blue.

### Measurement of paraprotein level

Human paraprotein secretion was analyzed as an indirect indicator of tumor burden. Serum samples were acquired from each mouse by tailbleed for the measurement of human Ig light chain in murine serum using enzyme linked immunosorbent assay (Human Kappa ELISA Kit; Bethyl Laboratories Inc). The assay was carried out as per the manufacturer’s protocol. At the end of the experiment, detection of kappa light chain in plasma of BM by ELISA was used to analyze the growth of tumor cells in mice. The result was measured by the absorbance (450 nm) in a microtiter plate reader, and the Ig light chain content was determined using the CurveExpert 1.4 software.

### Flow cytometry assay

The following antibodies were used to stain cells: Anti-Human CD138 (MI15, 552026), Anti-Human CD47 (B6H12, #556046), Anti-Human PD-L1 (MIH1, #557924), Anti-Human CD3 (SK7, #557832), were all purchased from BD Pharmingen. Anti-human IFN-γ (4S.B3, #502506) antibody and Brefeldin A Solution were purchased from BioLegend. Flowcytometry analyses were performed on FACS CantoII. Data were analyzed with Flow Jo Software.

### Cell cycle and apoptosis analysis

Cell cycle analysis was performed by fixing cells with 70% ice-cold ethanol for 3 hours, treating with 100 μg/ml RNase A, and staining with 50 μg/ml propidium iodide for 20 minutes. For the apoptosis analysis, cells were incubated with Annexin V and 7-Amino-Actinomycin D (BD Biosciences). Data were collected on a BD Canto II Flow Cytometer and analyzed with FlowJo software (7.6.1).

### RNA extraction and quantitative real-time PCR

Total RNA was extracted using TRIzol reagent (Invitrogen, USA). Reverse transcription of miRNA or mRNA was conducted with All-in-OneTM miRNA RT-qPCR Detection kit (GeneCopoeia, QP015) or All-in-OneTM First-Strand cDNA Synthesis kit (GeneCopoeia, AORT-0060). To quantify the miR-15a expression or mRNA expression of DNp73, MYC, MYCN, p65, CDK7 and CD47, real-time PCR was performed on Quant Studio 5 (Thermo) using All-in-One™ miRNA RT-qPCR kit (GeneCopoeia, QP015) or SYBR Premix Ex Taq™ (Takara, RR42LR). PCR primers for U6 (HmiRQP9001) and miR-15a (HmiRQP0223) were all purchased from GeneCopoeia. In each RT-qPCR, snRNA U6 and GAPDH mRNAs were used as control for normalization respectively. The relative expression was calculated using the 2-ΔΔCt method. PCR primer sequences were listed as follows:

MYC-F: 5’- CTGCGACGAGGAGGAGAACT-3’, MYC-R: 5’-GGCAGCAGCTCGAATTTCTT-3’;

MYCN-F: 5’-ACCCGGACGAAGATGACTTCT-3’, MYCN-R: 5’- CAGCTCGTTCTCAAGCAGCAT -3’;

CD47-F: 5’-GGCAATGACGAAGGAGGTTA-3’, CD47-R: 5’- ATCCGGTGGTATGGATGTGA -3’;

PD-L1-F: 5’- GGCATTTGCTGAACGCAT-3’, PD-L1-R: 5’-CAATTAGTGCAGCCAGGT-3’;

CDK7-F: 5’- ATGGCTCTGGACGTGAAGTCT -3’, CDK7-R: 5’- GCGACAATTTGGTTGGTGTTC -3’;

DNP73-F: 5’-ACTAGCTGCGGAGCCTCTCCC-3’, DNP73-R: 5’-TGCTCAGCAGATTGAACTGG-3’;

GAPDH-F: 5’-GAAGGTGAAGGTCGGAGTC-3’, GAPDH-R: 5’-GAAGATGGTGATGGGATTTC-3’;

U6:5′ -TGCGGGTGCTCGCTTCGGCAGC-3′, 5′−CCAGTGCAGGGTCCGAGGT -3′

### Immunofluorescence staining

Immunofluorescence was performed as previously described. MM cells were spun down on glass slides and then fixed in -20°C acetone/methanol (1:1 volume) for 10 min. Cells were then incubated with primary antibody against Phospho- Histone H2A.X or CD47 (1:100 dilution in PBS containing 4% bovine serum albumin, CST) overnight at 4°C and incubated with the secondary antibodies Alexa Fluor 488-conjugated goat anti-mouse immunoglobulin G (IgG; H+L; 1:200, Invitrogen) or Alexa Fluor 568-conjugated goat anti-rabbit IgG at room temperature for 30 min in the dark. Nuclei were stained with DAPI. Microscopic images were captured using an Ultra VIEW Vox confocal microscope (Perkin Elmer, USA), and analyzed with Image J software.

### RNA-seq and GSEA analysis

RNA was extracted using the Qiagen RNeasy Kit. Library construction and RNA sequencing were performed on a BGISEQ-1000 system by Beijing Genomic Institution (BGI, China). Gene-set enrichment analysis (GSEA) was performed with GSEA_4.0.3 (gene set enrichment analysis, Broad Institute).

### ChIP assay

ChIP assays were performed using a Thermo Scientific Pierce Magnetic ChIP Kit (#26157) in accordance with the manufacturer’s protocol. Myeloma cells were cultured in RPMI-1640 medium containing 10% FBS for 24 hours. Crosslinking was achieved using a final concentration of 1% formaldehyde in the media for 10 minutes. After sonication, 1% of the soluble chromatin fraction was de-cross-linked at 4 °C overnight and used as input. The remaining chromatin fraction was immunoprecipitated with DNp73 antibody and de-cross-linked. DNA was purified using the QiaQuick PCR purification kit (Qiagen, Venlo, Netherlands) and analyzed by PCR. Purified DNA was used in RT-qPCR for the quantification of protein–DNA binding. The primer sequences were as follows:

DNp73-P-F: 5′ CCTCTGCCACAGGAAGAGAC 3′, DNp73-P-R: 5′ GCTGAGGACGAAAGGACGAT 3′;

MYCN-P-F: 5′AGGCTGGGTCTCAGGAGGTG 3′, MYCN-P-R: 5′CTGGCTCTGCTTCCTAGGGG 3′.

### Cell migration and invasion assay

Cell migration and invasion were determined by transwell assay. Briefly, transfected ARP1 and 8226 cells in serum-free RPMI-1640 were added to the upper chamber with or without matrigel. Then, RPMI-1640 containing 20% FBS was added to the lower chamber as a chemoattractant. After incubation for 24 h, cells on the upper surface were removed, and cells attached to the bottom were fixed with methanol and stained with 0.5% crystal violet for 30–60 min. The cells of five randomly selected fields were counted using an inverted microscope.

### CRISPR-Cas9 knockout of DNp73

MM cell lines were stably infected with lentiviral LentiCRISPR v.2 expressing CAS9 gene and sgRNAs targeting DNp73 or control lentivirus, and selected using puromycin antibiotic, 10 μg ml−1. DNp73 knock-out was validated by western blotting. Three guide sgRNAs were designed using the CHOPCHOP web tool to target the following sequences:

sgRNA-DNp73-1:5′ GCTCCAGAGGTGCTCAAACGTGG 3′,

sgRNA-DNp73-2:5′ AGGTCGAAGTAGGTGCTGTCTGG 3′,

sgRNA-DNp73-3:5′ GGGCGGAACGGATTCCAGCATGG 3′,

sgRNA-DNp73-4:5′ TCAAACGTGGTGCCCCCATCAGG 3′.

### Statistical analyses

The quantitative results are presented as the mean ± standard deviation (SD) from ≥3 independent experiments. Statistical analysis was performed using GraphPad Prism (version 8.01, GraphPad Software Inc.). For survival analysis, the log-rank (Mantel-Cox) test was performed. All other analyses used the Student *t* test. *p<0.05, **p<0.01, ***p<0.001, and ****p<0.0001.

## Results

### Overexpression of DNp73 causes by the miRNA-15a/NF-κB pathway in MM cells

**Figure 1:**
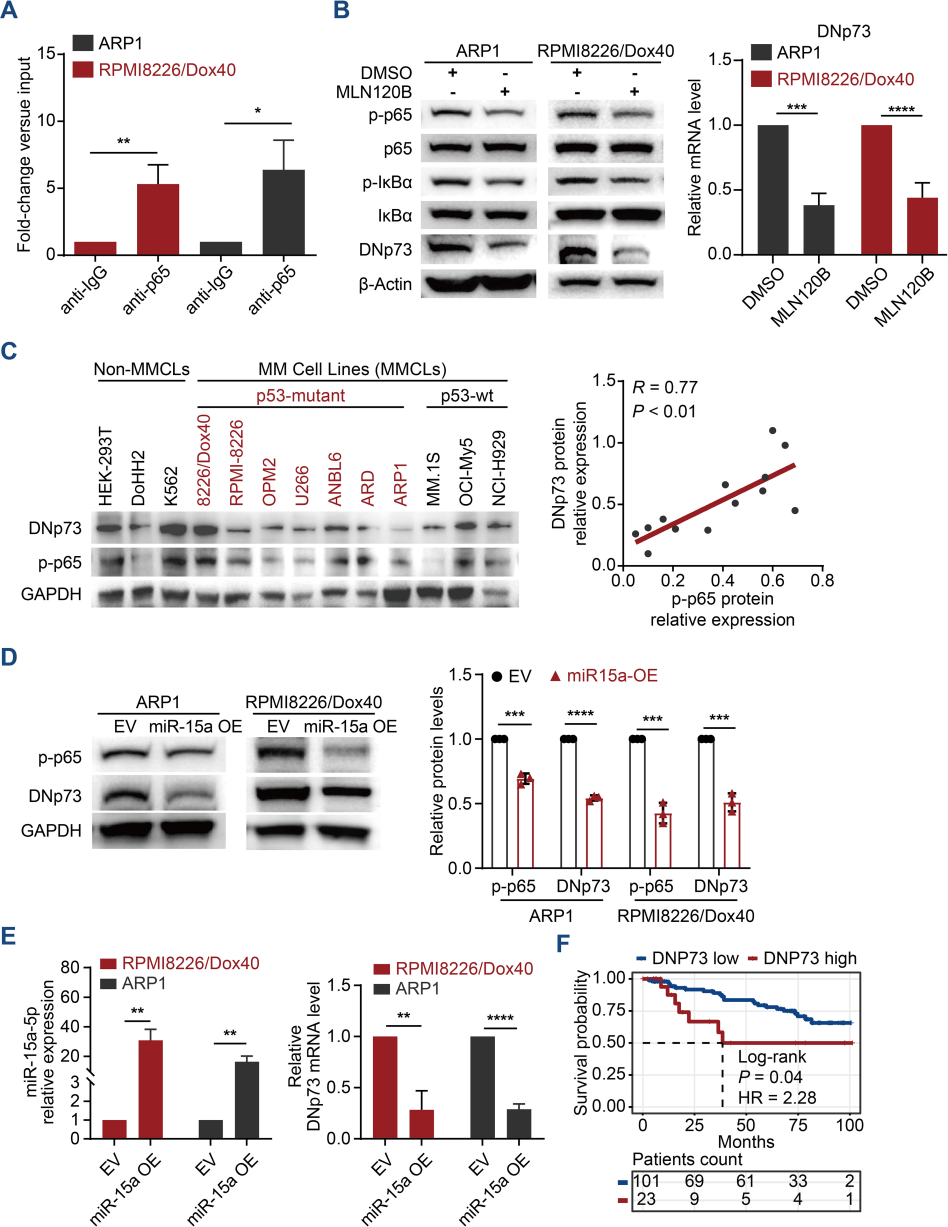
DNp73 is upregulated via the miRNA-15a/NF-κB pathway in MM cells. **(A)** ChIP−qPCR was performed to identify the binding between p65 and DNp73 in MM cells. **(B)** ARP1 and RPMI 8226/Dox40 cells were cultured with MLN120B (20 μM) or DMSO. After 2 h of treatment, total RNA and whole cell lysates were extracted and subjected to quantitative real time PCR and immunoblot analysis. **(C)** Immunoblotting detection of DNp73, phosphorylated p65 (p-p65) and GAPDH levels in multiple MM cell lines and non-MM cell lines. The scatter plot (right) shows that p-p65 expression was positively correlated with DNp73 expression in 13 cell lines (r = 0.77, P < 0.01). **(D)** The protein levels of p-p65 and DNp73 were detected in miR-15a-overexpressing and control ARP1 and RPMI8226/Dox40 cells. **(E)** RT−qPCR analysis revealed the expression of miR-15a and DNp73 in miR-15a-overexpressing and control ARP1 and RPMI8226/Dox40 cells. **(F)** Kaplan−Meier analysis was performed in MM patients with high levels of DNp73 based on the cancer genome atlas MMRF-CoMMpass cohort. Data are presented as mean ± SD of ≥3 independent experiments, and comparisons were evaluated by using the 2-tailed Student t test. *P <.05, **P <.01, ***P <.001, ****P <.0001.

The NF-κB pathway is one of the most crucial pathways in multiple myeloma, not only due to its role in pathogenesis but also its significance in various treatment strategies (26, 27). Our previous studies revealed that miRNA-15a is a tumor suppressor involved in the aggressive proliferation and drug resistance of MM cells (17). Here, we further reveal that miRNA-15a downregulation in MM cells activates NF-κB-p65, which promotes the expression of DNp73 and involves in the pathogenesis of MM for the first time. The cis-regulatory elements in the DNp73 promoter were predicted using publicly available chromatin immunoprecipitation sequencing (ChIP-seq) data from ReMap2022 in the UCSC Genome Browser (https://genome.ucsc.edu/) (**Supplementary Figures 1A, B**). Referring to the binding signals reported (GSE75562. RELA. HEK293) and the consensus sequence of NF-κB-p65 (28), we designed specific qPCR primers and a ChIP-qPCR assay was conducted to determine the enrichment of the NF-κB-p65 factor in the DNp73 gene promoter region. As **Figure 1A** showed, the DNp73 promoter region was significantly enriched in the NF-κB-p65 fraction obtained in a pulldown assay with ARP1 and RPMI 8226/Dox40 myeloma cell lines. Further, we treated MM cells with MLN120B (NF-kB inhibitor), and the expression of DNp73 was notably decreased both in protein and transcriptional level (**Figure 1B**) in accordance with the inhibition of p65 phosphorylation (activation). And immunoblots analysis showed that the DNp73 level was positively correlated with the activity of NF-κB-p65 (the phosphorylation of p65, p-p65) in MM cell lines (**Figure 1C**). However, no correlation was found between DNp73 levels and TP53 mutation status (**Figure 1C**, **Supplementary Figure 1C**).

According to our previous study that miR-15a was downregulated in MM cells and correlated with poor outcomes for MM patients (29). Here, we further demonstrated that NF-κB-p65 activation is inhibited in miRNA-15a-overexpressing MM cells and that miRNA-15a upregulation leads to the downregulation of DNp73 at both the transcriptional and protein levels (**Figures 1D, E**). Analysis of MM patient samples confirmed the negative correlation between the levels of miRNA-15a and DNp73 (**Supplementary Figure 1D**). Additionally, we did not find a negative correlation between the two isoforms of TP73 (TAp73 and DNp73) in MM cells (**Supplementary Figures 1E, F**). Kaplan-Meier analysis in the CoMMPASS dataset of MM patients indicated that the patient with high-level of DNp73 has inferior outcome compared with the low-level patients (**Figure 1F**). These data indicated that DNp73 was upregulated in MM cells mediated by the miRNA-15a/NF-κB-p65 signaling pathway. More importantly, DNp73 levels were not associated with TP53 status or TAp73 levels.

### DNp73 protects MM cell growth against DNA damage agent induced cell death

**Figure 2:**
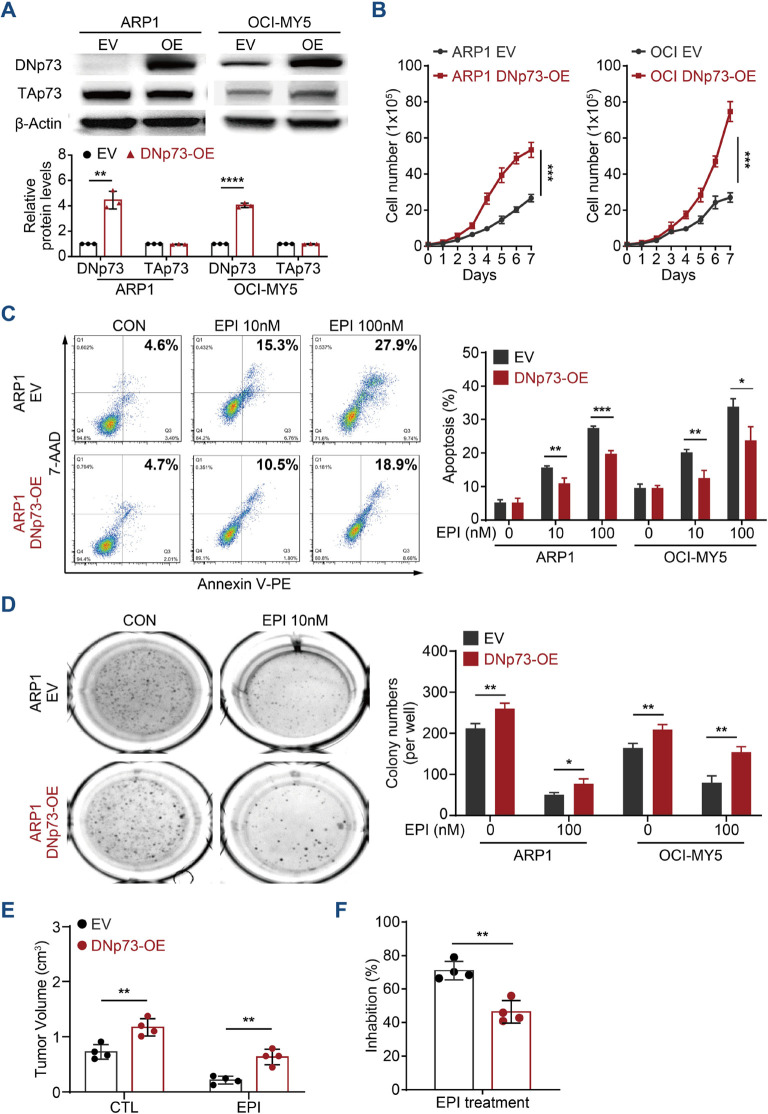
Enhanced DNp73 expression promotes MM cell growth and drug resistance. **(A)** Immunoblotting detection confirmed the overexpression of DNp73 in ARP1 and OCI-MY5 cell lines. **(B)** Cell proliferation was detected by absolute cell counting. **(C)** Cell apoptosis was evaluated in MM cells treated with DMSO and the DNA damage agent epirubicin (EPI). A representative diagram of the flow cytometry results is shown on the left. The statistical analysis data are shown on the right. **(D)** A colony formation assay was conducted with equal numbers of MM cells seeded in semisolid methylcellulose complete medium with or without EPI (100 nM) (left). The histograms indicate the absolute number of colonies formed (right). Representative plates and the manual quantification of colonies made from five independent plates. **(E, F)** An *in vivo* study was performed to confirm the proliferation capacity of MM cells with high levels of DNp73 in NOD/SCID mice. Tumor volume and drug inhibition rate were assessed (n = 4). The data are displayed as the mean plus SD of three replicates, and statistical significance was calculated and presented as the P value. *P <.05, **P <.01, ***P <.001, ****P <.0001.

According to reports, DNp73 functions as an oncogene (30, 31). Therefore, we first investigated the roles played by DNp73 in MM cell proliferation. Immunoblots analysis validated the overexpression of DNp73 in both TP53 wild-type OCI-My5 MM cells and TP53 deletion ARP1 cells (**Figure 2A**). As the data showed, there was no correlation between the level in TAp73 and DNp73 in MM cells. Flow cytometry analysis showed an accelerated cell cycle in DNp73-OE cells (**Supplementary Figure 2A**), which promoted MM cell proliferation compared to that in control cells (**Figure 2B**). To explore whether resistance in MM cells following irradiation-induced apoptosis is mediated by DNp73 overexpression, and is accompanied by enhanced recovery after radiation-induced DNA damage, changes in DNA damage response in irradiated MM cells were analyzed by immunofluorescence. Compared to that in empty vector (EV) control cells, the number of γH2AX-positive foci was decreased after irradiation, and apoptosis induced by irradiation was also decreased in DNp73-OE MM cells compared to control group (**Supplementary Figures 2B, C**). These data demonstrated that DNp73 overexpression protected MM cells against the DNA damage-induced apoptosis. The DNA damage agent epirubicin (EPI) combined with bortezomib and dexamethasone (PAD) is the National Comprehensive Cancer Network recommendation for primary MM therapy. Here, we found that DNp73-OE cells were resistant to EPI-induced cell death as well. The apoptosis rate was significantly decreased in DNp73-OE ARP1 and OCI-MY5 MM cells (**Figure 2C**, **Supplementary Figure 2D**). Furthermore, after exposure to genotoxic drugs melphalan or bendamustine for 24 hours, apoptosis rates were lower in DNp73 OE cells compared to control cells (**Supplementary Figure 2E**). These results confirmed that DNp73-OE promoted MM cells survival via against the genotoxic agent induced cell death. A soft agar-based colony formation assay further indicated that DNp73-OE MM cells were more resistant to EPI-induced cell death (**Figure 2D**, **Supplementary Figure 2F**). We next confirmed the increase in the growth of the DNp73-OE MM cell line implanted in a xenograft mouse model (**Supplementary Figures 2G, H**). Consistently, overexpression of DNp73 in MM cells led to an increase in tumor volume and induced drug resistance in MM cells (**Figures 2E, F**).

**Figure 3:**
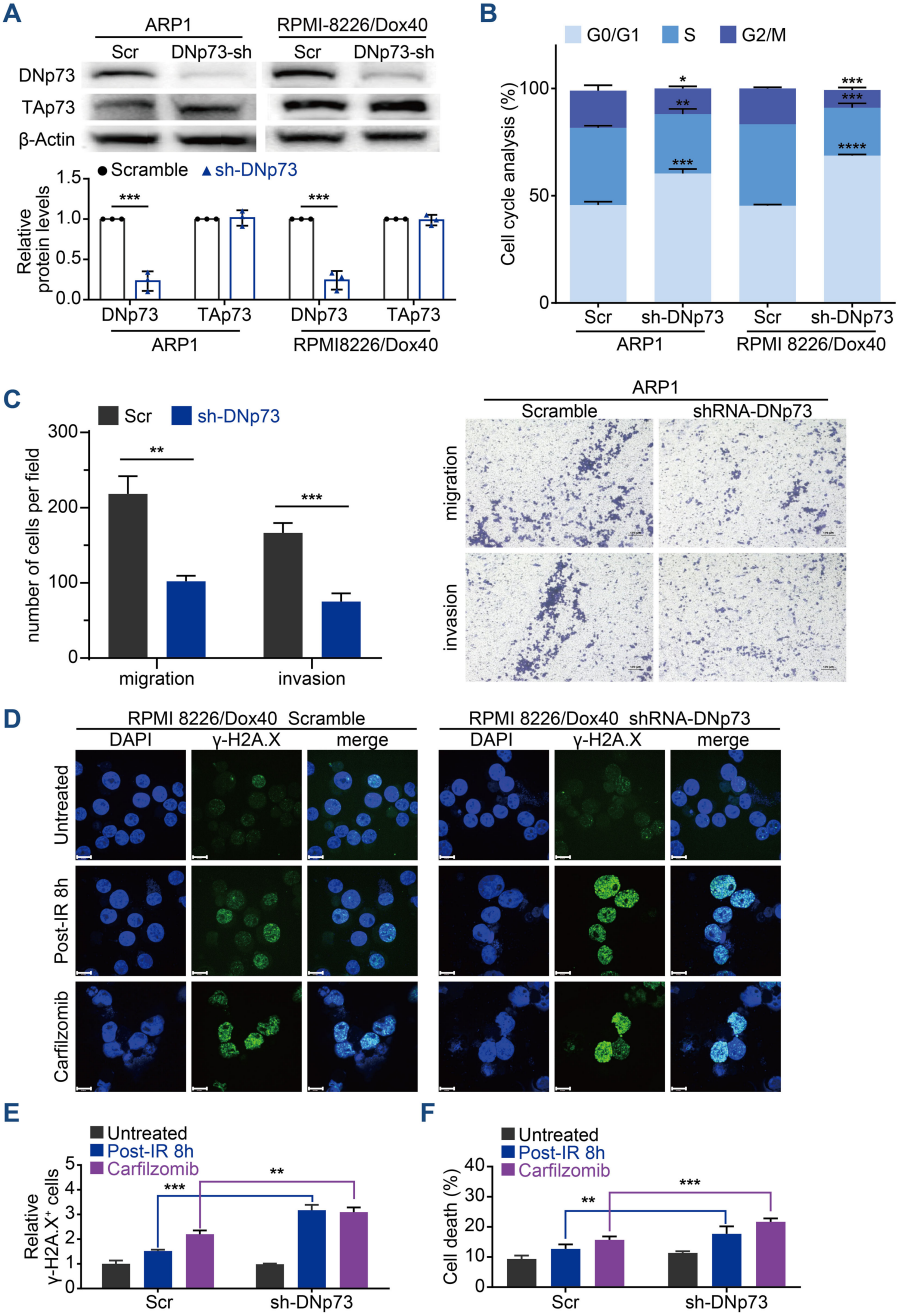
Knockdown of DNp73 inhibits the myeloma cell cycle, metastasis and DNA damage repair. **(A)** Knockdown of DNp73 in ARP1 and RPMI 8226/Dox40 cells was confirmed by immunoblotting. **(B)** Flow cytometry analysis of the cell cycle in ARP1 and RPMI 8226/Dox40 scramble or shRNA-DNp73 cells. **(C)** The migration and invasion abilities of scramble and shRNA-DNp73 cells were measured by Transwell assay. **(D, E)** Response to radiation or carfilzomib in RPMI 8226/Dox40 cells. γH2AX foci were counted 8h after irradiation, or 24 hours treatment with carfilzomib. Cells were immunostained with anti-γH2AX (green) and DAPI (blue) and analyzed using confocal microscopy. Graphic presentation of the relative γH2AX+ Scramble and shRNA-DNp73 cells. The cells containing > 5 γH2AX foci were recorded as γH2AX+ cells, whose percentage was averaged from at least 100 cells. The number of γH2AX foci without irradiation was normalized to 1. Scale bars, 14 µm. **(F)** The RPMI 8226/Dox40 cells were exposed to radiation or carfilzomib and the percentage of surviving cells were assayed by trypan blue exclusion. The data are displayed as the mean plus SD of three counts, and statistical significance was calculated and represented as the P value. *, P < 0.05; **, P < 0.01; ***, P < 0.001, ****P <.0001.

To confirm the effects of DNp73 on MM cell proliferation, doxycycline-inducible DNp73-knockdown MM cell lines were constructed via a lentivirus delivery system. To prevent TP53 disruption of DNp73 function, TP53 gene deletion cell line (ARP1 cells) and TP53 gene mutation (RPMI 8226/Dox40) MM cells (doxorubicin-resistant) were utilized. After treatment with 2 μg/mL doxycycline, the knockdown efficiency was confirmed by immunoblotting (**Figure 3A**). Flow cytometry analysis showed that DNp73 knockdown induced cell cycle arrest at the G0/G1 phase (**Figure 3B**, **Supplementary Figure 3A**) in both TP53-deleted and TP53-mutated MM cell lines. Reducing DNp73 expression efficiently abrogated MM cell proliferation (**Supplementary Figure 3B**). The levels of cell cycle-associated proteins, including the cyclin-dependent kinases CDK4, CDK6, cyclin D3 and pho-Rb, were reduced, but the levels of the cell cycle inhibitors p21 and p27 were increased following DNp73 knockdown in both TP53-deleted and TP53-mutated MM cells (**Supplementary Figure 3C**). More importantly, a transwell migration assay showed that the numbers of migrating and invading MM cells were significantly reduced by DNp73 knockdown (**Figure 3C**, **Supplementary Figure 3D**). Knockdown DNp73 expression significantly increased DNA damage after exposure to irradiation or DNA damage agents, including EPI, Pomalidomide, Carfilzomib, and MM cells showed increased sensitivity to radiation-induced cell death (**Figures 3D–F**, **Supplementary Figure 3E**). To further confirm these results, the DNp73 was knockout by CRISPR-Cas9 system in MM cell lines (**Supplementary Figures 3F, G**). Exposure to the DNA damage reagent calfilzomib or irradiation, compared to that in control cells, the number of apoptosis was significantly increased in DNp73-KO MM cells via induced significant DNA damage response (**Supplementary Figures 3H, I**). These data support that DNp73 protects MM cell growth against DNA damage reagent induced cell death.

### DNp73 induces multidrug resistance in MM

**Figure 4:**
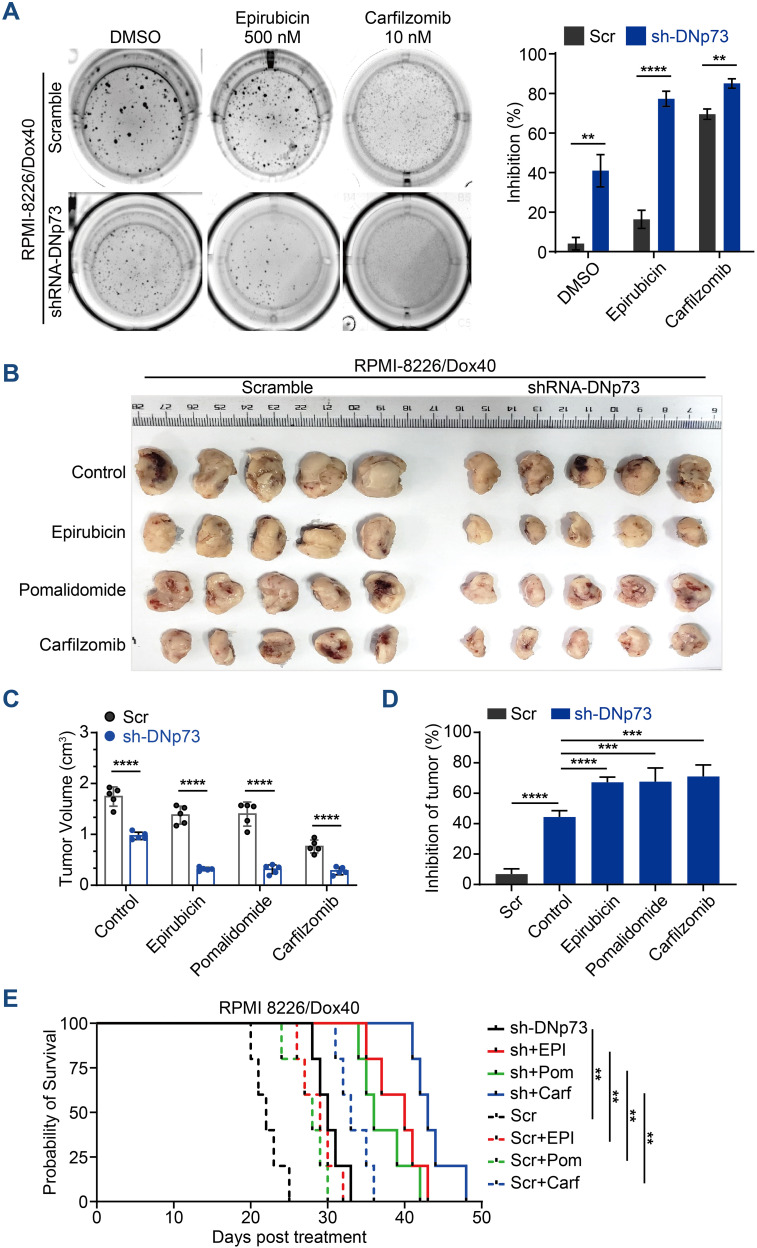
Knockdown of DNp73 inhibits myeloma cell growth and sensitizes cells to chemotherapeutic drugs. **(A)** A colony formation assay was utilized to quantify the colony formation of MM cells after coculture with or without epirubicin or carfilzomib for 14 days. The inhibition rate of colonies is shown on the right. **(B)** RPMI 8226/Dox40 shDNp73 cells were inoculated into NOD/SCID mice. shRNA expression was induced by doxycycline, which was administered every other day on the day after the injection of tumor cells. **(C)** Tumor volume assessment revealed that the mice in the doxycycline-induced shRNA DNp73 group had smaller tumors than those in the control group (n = 5). **(D)** The drug inhibition rate of each group was analyzed. Bars represent the means ± SD each group. **P <.01, ***P <.001, ****P <.0001. **(E)** Survival analysis was performed for the four groups. Kaplan−Meier analysis showed that knockdown of DNp73 significantly extended the survival of tumor-bearing mice compared with the control group. The significance of differences was analyzed by the log-rank Mantel–Cox test. All comparisons of survival curves resulted in P values < 0.01.

In addition to promoting cell proliferation, DNp73 notably induced drug resistance in MM cells. DNp73 knockdown significantly increased the sensitivity of MM cells to multiple drug treatment, including the DNA damage agent epirubicin, the immunomodulatory agent pomalidomide and the proteasome inhibitor carfilzomib. Increased cell apoptosis was observed (**Supplementary Figure 3E**). Additionally, a soft agar-based colony formation assay confirmed DNp73 knockdown alone inhibited MM cell proliferation (DMSO group), and the inhibition was more obvious after drug treatment (**Figure 4A**). Next, we investigated whether DNp73 was required for myelomagenesis and disease progression in a xenograft mouse model. The doxorubicin-resistant TP53-mutated RPMI 8226/Dox40 MM cell line was utilized. Consistent with the *in vitro* study, the subcutaneous tumor volume in the DNp73-knockdown mouse group was significantly decreased compared with that in the control group (**Figures 4B, C**, **Supplementary Figures 4A, B**). Moreover, an *in vivo* study confirmed increased sensitivity to epirubicin, pomalidomide and carfilzomib in DNp73-knockdown cells (**Figure 4D**). These data support that downregulation of DNp73 significantly increases the inhibition of tumor growth induced by the indicated treatment. The potent suppression of tumor growth in the DNp73-knockdown group translated to a significant increase in survival time compared to that in the scramble group (**Figure 4E**).

### DNp73 activated the MYC signaling pathway

**Figure 5:**
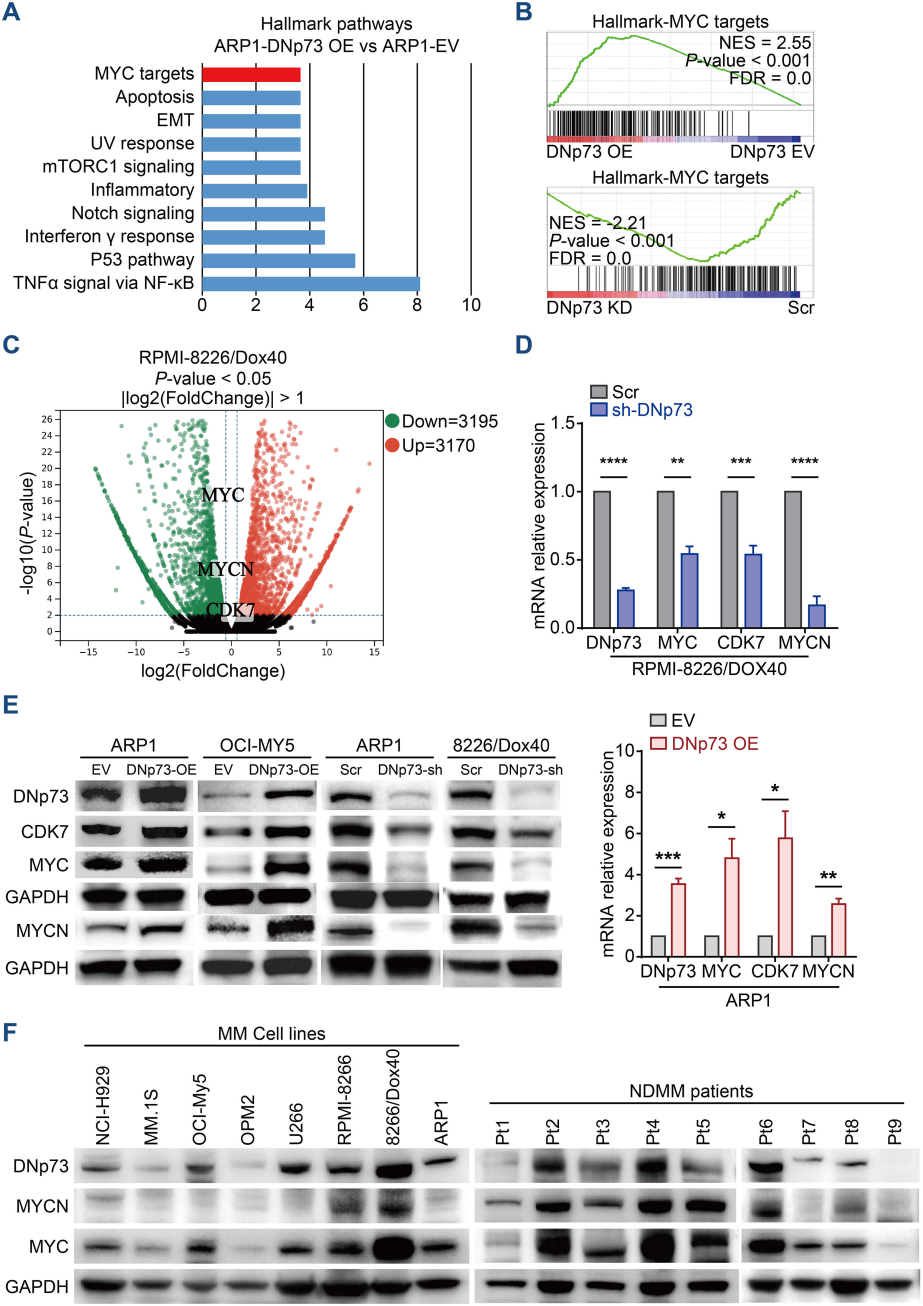
RNA-seq indicates that DNp73 activates the MYC-related pathway. **(A)** GSEA showing the enriched gene signature associated with pathways in ARP1 cells upon DNp73 overexpression. **(B)** GSEA showed that DNp73 expression was positively correlated with MYC target-related genes in ARP1 cells. **(C)** Volcano plots of normalized gene expression in RPMI 8226/Dox40 cells were analyzed by RNA-seq. **(D, E)** Detection of DNp73, CDK7, MYC, and MYCN expression in MM cells using RT−qPCR and Immunoblotting. **(F)** Whole-cell lysates of MM cell lines and MM patient samples were subjected to immunoblot analysis with the indicated antibodies. Data are presented as mean ± SD of ≥3 independent experiments, and comparisons were evaluated by using the 2-tailed Student t test. *P <.05, **P <.01, ***P <.001, ****P <.0001.

To clarify the pathways involved in DNp73-mediated myeloma cell survival and drug resistance, we performed bulk RNA-seq on ARP1, OCI-My5, and RPMI 8226/Dox40 cells with aberrant DNp73 expression (**Supplementary Figure 5A**). Gene set enrichment analysis (GSEA) showed that signaling pathways of MYC targets and DNA repair were positively enriched in DNp73-OE MM cells and that the p53 pathway was negatively enriched in these cells (**Figures 5A, B**, **Supplementary Figure 5B**). These data suggest that DNp73 promotes myeloma cell survival through multiple signaling pathways. Notably, the levels of the oncogenes MYC and MYCN, which play important roles in the pathogenesis of cancer, were reduced in DNp73-knockdown MM cells, as shown in **Figure 5C**. In addition, the CDK7 gene, which directly regulates MYC expression, was downregulated in DNp73-knockdown cells as well. Our RT-qPCR and immunoblot assays further confirmed that MYC, MYCN, CDK7 were expressed at lower levels in DNp73-knockdown cells than in control cells, suggesting that the downregulation of the oncogene MYC was increased after the downregulation of CDK7 expression in cells with reduced DNp73 expression; in contrast, DNp73 overexpression promoted the expression of MYCN, MYC and CDK7 (**Figures 5D, E**, **Supplementary Figures 5C, D**). The correlation between DNp73 and MYCN or MYC protein expression was further examined in 8 MM cell lines and 16 primary patient samples via immunoblot assay. The results indicated that the level of DNp73 was positively correlated with the level of MYCN or MYC, in both MM cell lines and primary patient samples (**Figure 5F**, **Supplementary Figures 5E, F**). In DNp73 KO MM cell lines, the MYCN and MYC decreasing was confirmed (**Supplementary Figure 5G**).

### DNp73 regulates MYCN expression at transcriptional level

**Figure 6:**
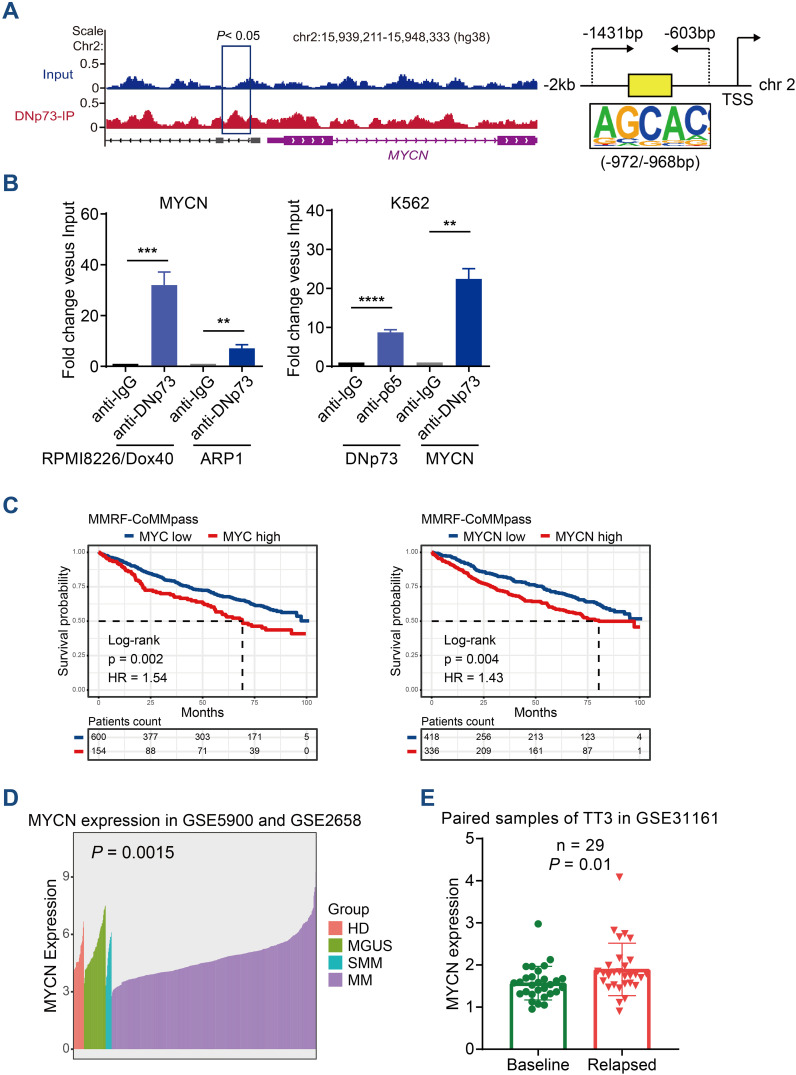
ChIP-seq analysis identified that DNp73 acts as a transcription factor to regulate MYCN expression. **(A)** ChIP-seq assay of DNp73 binding to the MYCN promoter. Schematic diagram of the binding site for DNp73 and the promoter region of MYCN located 2 kb upstream of the TSS. **(B)** DNp73 ChIP−qPCR for the MYCN promoter in RPMI 8226/Dox40, ARP1 and K562 cells. Data are presented as mean ± SD of 3 independent experiments. **P <.01, ***P <.001, ****P <.0001. **(C)** Kaplan−Meier analysis was performed in MM patients with high levels of MYCN or MYC based on the cancer genome atlas MMRF-CoMMpass cohort. **(D)** The MYCN expression in the GSE5900 and GSE2658 dataset. The level of MYCN was increased in NDMM patients compared with HD, MGUS and SMM. **(E)** The MYCN expression in the GSE31161 dataset. MYCN level was increased in relapsed patient samples.

DNp73 can activate target genes, and we hypothesized that it regulates MYC or MYCN expression at the transcriptional level. Next, we performed a ChIP-seq assay with DNp73 to determine the binding sites in the RPMI 8226/Dox40 cell line. Combined ChIP-seq and RNA-seq data, the target genes regulated by DNp73 in MM cells were identified (**Supplementary Figure 6A**). As expected, MYCN was one of the most significant genes detected by ChIP-seq in RPMI 8226/Dox40 cells (-log10 P value=3.15), and a significant expression fold change was confirmed with RNA-seq data. The motifs most frequently bound by the DNp73 transcription factor were identified by ChIP-seq, and they were shown in **Supplementary Figure 6B**. DNp73 bound to the promoter region of MYCN located upstream of the TSS coding for MYCN on chr.2 (**Figure 6A**). According to the motifs identified, ChIP−qPCR was performed to confirm DNp73 occupation of the MYCN promoter in RPMI 8226/Dox40, ARP1 MM cells, and in a non-MM cell line K562 (**Figure 6B**). Furthermore, Kaplan-Meier analysis in CoMMPass MM patient cohort showed that MM patients with high MYC or MYCN expression exhibited worse outcomes than those with low expression with the best expression signal cutoff value determined using the R package (**Figure 6C**). According to our data in MMRF-CoMMPass dataset, 20.4% (154/754) of MM patients with elevated MYC expression showed lower overall survival (OS) (hazard ratio, HR=1.54, P=0.002, log-rank test). In addition, 44.6% (336/754) of the MM patients with elevated MYCN expression showed shorter OS (HR=1.43, P=0.004, log-rank test). To confirm the expression of MYCN in MM patient samples, we analyzed the MYCN expression in the GSE5900, GSE2658 and GSE31161 dataset. The results showed that the level of MYCN in patients with NDMM was higher than that in patients with HD, MGUS, and SMM (**Figure 6D**). Notably, MYCN level was further increased in relapsed patient samples compared with the baseline level (**Figure 6E**).

### DNp73 facilitates T-cell dysfunction via the upregulation of PD-L1 on MM cells

**Figure 7:**
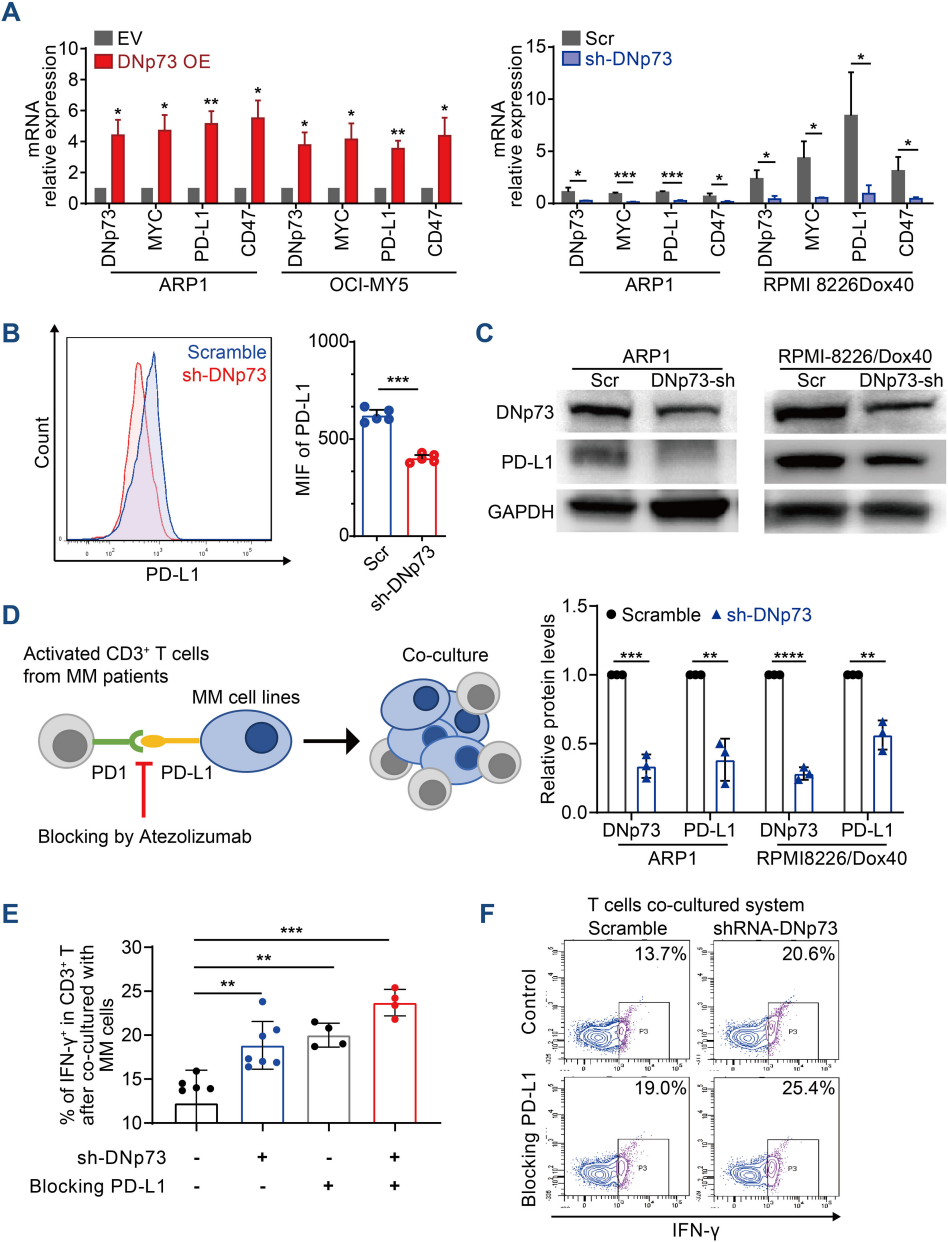
DNp73 promotes T-cell dysfunction via upregulation of PD-L1 on MM cells. **(A)** RT−qPCR of DNp73, MYC, PD-L1 and CD47 expression in MM cells. **(B, C)** The protein level of PD-L1 in scramble and shRNA DNp73 cells through flow cytometric analysis and immunoblotting. **(D–F)** The levels of the cytotoxic cytokine IFN‐γ were detected by flow cytometry analysis in CD3^+^ T cells. Data are presented as mean ± SD of ≥3 independent experiments, and comparisons were evaluated by using the 2-tailed Student t test. *P <.05, **P <.01, ***P <.001, ****P < .0001.

Immune evasion is a critical characteristic of tumor cells. In addition to clonal evolution, the immunosuppressive tumor microenvironment plays pivotal roles in MM patient survival and disease progression (32). According to a report by Casey et al., MYC regulates the expression of two immune checkpoint proteins on the tumor cell surface: the innate immune regulator CD47 and the adaptive immune checkpoint PD-L1 (33). In addition, Melaiu et al. reported that MYC and MYCN controlled the expression of PD-L1 in neuroblastoma cells both *in vitro* and *in vivo* (34). Here, we showed that the mRNA levels of CD47 and PD-L1 were increased in MM cells with high DNp73 expression compared with control cells (**Figure 7A**). Moreover, both flow cytometry analysis (**Figure 7B**) and immunoblot assays (**Figure 7C**) indicated that PD-L1 level was decreased in DNp73-knockdown MM cells. These data suggest that DNp73 facilitates the immune evasion of MM cells by upregulating the expression of PD-L1 on MM cells. To confirm this hypothesis, a coculture experiment with MM cells and T cells was performed (**Figure 7D**). CD3-positive T cells were isolated from bone marrow biopsy samples of MM patients and cocultured with anti-CD3/CD28 beads. Then, MM cells were added to compare the effector functions of T cells via the detection of IFN-γ production. Our data showed that the expression of interferon (IFN)-γ in T cells was significantly increased after coculturing with DNp73-knockdown MM cells compared with that in scramble controls. In addition, T-cell activation was also enhanced after the PD-L1/PD-1 signaling pathway was blocked by treatment with the PD-L1 monoclonal antibody atezolizumab (**Figures 7E, F**).

### DNp73 suppresses phagocytosis promoting the immune evasion of myeloma cells

**Figure 8:**
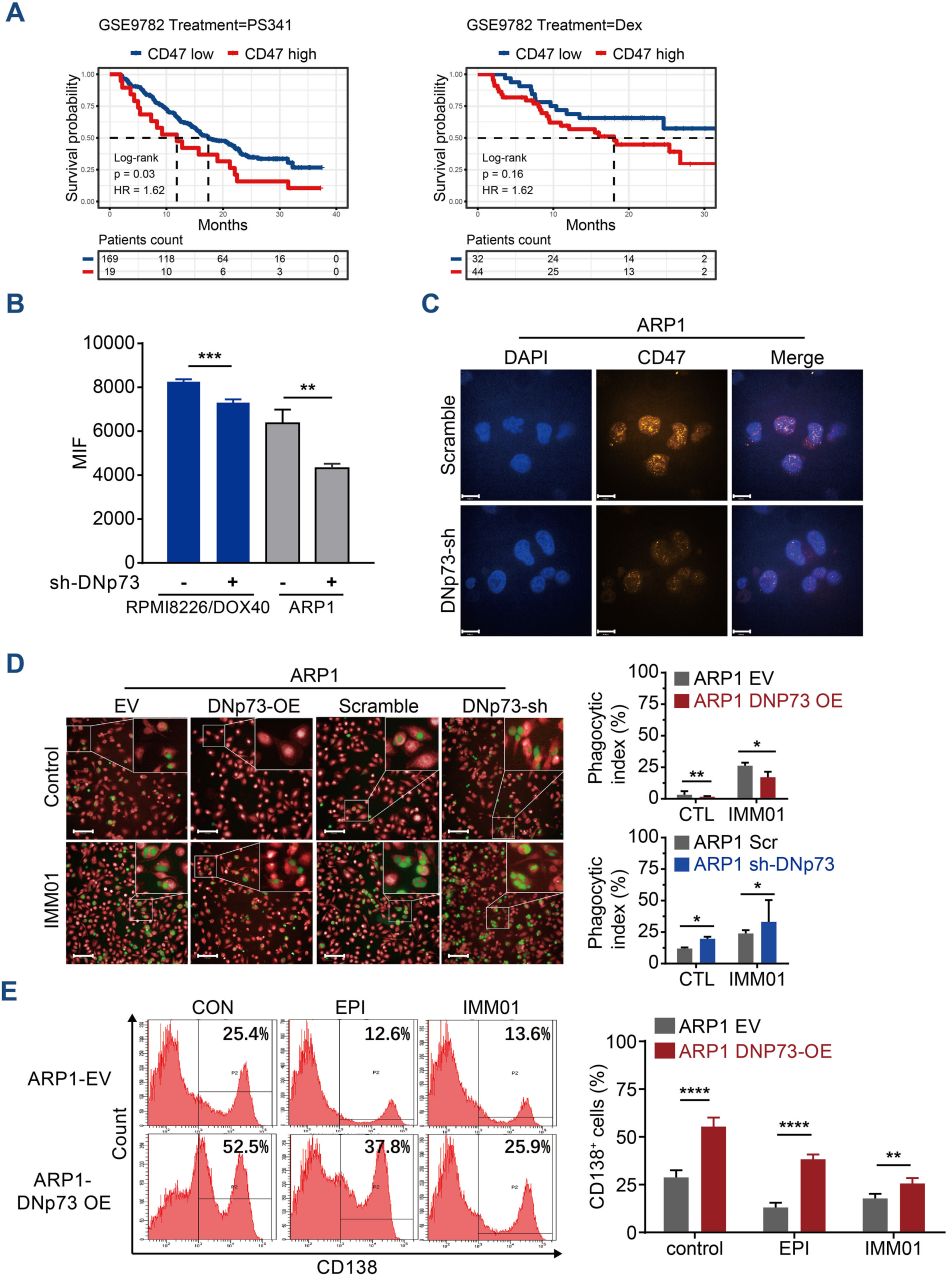
Anti-CD47 treatment with IMM01 overcomes DNp73-induced immune evasion in MM. **(A)** Kaplan−Meier analysis was performed in MM patients with high levels of CD47 based on the GEO data of individuals with relapsed MM (GSE9782). **(B)** The protein level of CD47 on myeloma cells was examined through flow cytometric analysis. **(C)** Cells were immunostained with anti-CD47 (orange) and DAPI (blue) and analyzed using confocal microscopy. Scale bars, 14 µm. **(D)** Phagocytosis of various myeloma cells was detected. The experimental procedure is shown on the left. Green signals indicate macrophages engulfing CFSE-labeled myeloma cells, and red signals represent macrophages. A representative image of red macrophages that the green myeloma cells are inside, quantitation of the phagocytic index of myeloma cell lines as described in Materials and Methods. Scale bars, 100 µm. **(E)** Tumors were allowed to establish for 10 days, after which mice were randomized into treatment groups. NOD/SCID mice (n =5/group) were treated for 3 weeks with DMSO, EPI or IMM01 (human anti-CD47 monoclonal antibody) according to the dosing schedules outlined in the Materials and Methods. Representative results of CD138^+^ human myeloma cell engraftment in mice treated with DMSO or drugs. Data are presented as mean ± SD of ≥3 independent experiments, and comparisons were evaluated by using the 2-tailed Student t test. *P <.05, **P <.01, ***P <.001, ****P <.0001.

CD47 has been reported to be highly expressed on MM cells, suggesting that blockade of the CD47 signaling pathway may be a potential therapy for MM (35). In this study, a Kaplan-Meier analysis based on the GSE9782 dataset of individuals with relapsed MM treated by Bortezomib (PS341) or Dexamethasone monotherapy indicated that a high-level of CD47 significantly correlated with a short survival time in drug-resistant and relapsed patients (**Supplementary Figure 7A**). The patients with high-level of CD47 have poor outcome in both Bortezomib monotherapy or Dexamethasone monotherapy (**Figure 8A**). To investigate the effects of DNp73 on MM cell immune evasion via upregulating CD47 expression, a novel anti-CD47 monoclonal antibody, IMM01, produced by our collaborator (36), was used to treat *in vitro* and xenotransplantation models. As a novel SIRPα-Fc fusion protein targeting the CD47/SIRPα pathway, IMM01 exhibited strong functional antitumor activity realized through phagocytosis by blocking the “do not eat me” signal. We analyzed the mean fluorescence intensity of CD47 on myeloma cells through flow cytometry and immunofluorescence analyses. The expression of CD47 in DNp73 OE cells was higher than that in control cells (**Figures 8B, C**, **Supplementary Figures 7B, C**). DNp73 overexpression efficiently protected MM cells against phagocytosis by macrophages; however, blocking DNp73 and the CD47 signaling pathway via IMM01 treatment notably increased the phagocytosis rate (**Figure 8D**, **Supplementary Figures 7D, E**). Next, a xenograft MM mouse model was utilized to confirm these results (**Supplementary Figure 7F**). Four weeks after injection of DNp73-OE MM cells, an increase in the CD138^+^ human myeloma cell population and kappa light chain levels in the bone marrow of mice was detected, indicating an increased tumor burden (**Figure 8E**, **Supplementary Figure 7G**). Of note, the proportion of CD138^+^ cells in the bone marrow of the IMM01-treated group notably decreased compared to that in both the DNp73-OE group and the EV control group. Consistent with the results with the subcutaneous xenograft mouse model, DNp73 overexpression enhanced tumor growth and reduced tumor sensitivity to immunotherapy.

## Discussion

The results of this study underscore two points: DNp73 is a direct target of NF-κB-p65, and DNp73 induces progression and immune evasion in myeloma cells by directly targeting MYCN and modulating the MYC pathway. The working model of this study is shown in **Supplementary Figure 8**. MM is a malignancy that is stratified in part by cytogenetic abnormalities, including the instability of chr. 1, which is also the chromosome where DNp73 is located. Jeffrey and colleagues demonstrated that the instability of chr. 1 induces novel copy number gains in regions harboring genes such as MYCN (37). Chromothripsis is a frequent event in hematopoietic neoplasms and a driver of multiple myeloma (38). MYC/MYCN is overexpressed in diverse human tumors undergoing chromothripsis (39, 40). The enforced expression of DNp73 may be an important clinical indicator for high-risk MM patients with copy number aberrations on chr. 1.

TAp73 and DNp73 have been reported to show both opposing and overlapping functions in tumorigenesis (41). However, we did not find a high ratio of DNp73/TAp73 in the myeloma cell lines, although an imbalance between DNp73 and TAp73 has been reported to possibly contribute to tumorigenesis and resistance to chemotherapy in several malignancies (42). Moreover, studies reported by different groups were not entirely consistent in showing TAp73 to be a classical tumor suppressor gene. Both TAp73 and DNp73 are often overexpressed in a variety of human cancers (43, 44). Kanaga and other groups provided evidence that TAp73 plays a role in supporting tumor cell proliferation and hence may explain why many human tumors co-overexpress both TAp73 and DNp73, exerting strong survival pressure (13, 45).

Several studies have demonstrated that DNp73 may alter gene expression in a p53-independent fashion. DNp73 without a TA domain transactivated the caspase-2S promoter in p53-null H1299 lung adenocarcinoma cells (6). A ChIP assay with H1299 cells demonstrated that DNp73 bound to the promoter of the proangiogenic VEGF-A gene and induced its expression (14). Moreover, as the p21/CDKN1A promoter was downregulated after DNp73 binding, the expression of DNp73 in a p53-null cell line reduced the mRNA levels of p21 (46). Consistent with that, our results showed that DNp73 regulated gene expression independent on p53 mutation. Hence, these findings support that DNp73 influences gene transcription not only by antagonizing p53 but also via an independent way.

Despite extensive research on DNp73 expression in various tumors, the mechanisms controlling its expression remain unclear. Based on our previous studies (17), we further clarified that the downregulation of miRNA-15a led to the activation of the NF-κB-p65 signaling that promotes DNp73 transcription in myeloma cells. These findings also indicate that MYC expression is under controlled with the miR-15a/NF-κB/DNp73 signaling pathway. Interestingly, MYC directly regulates the expression of miR-15a/16-1 (47) and plays critical roles in hematological malignancy development. Our study also revealed that miR-15a expression was downregulated in DNp73-OE MM cells (**Supplementary Figure 7H**). Therefore, MYC/miRNA circuits and their deregulation likely contribute to the oncogenic functions of MYC. Several studies reported that wild-type and mutant p53 have been found to play opposing roles in cancer cells. Mutant p53 efficiently enhances NF-κB activation in numerous cancers (48–50), which implies that leads to the enhanced expression of DNp73. However, our study did not find the correlation between p53 statues (mutation or wide-type) and NF-κB activation (pho-p65) in MM cell lines.

Our previous research and others have uncovered the interactions of MM cells with the bone marrow microenvironment played pivotal roles in aggressive growth of MM cells and inducing the bone disease (18, 51, 52). This study further identified that the protein expression of the immune checkpoints CD47 and PD-L1 was notably upregulated in DNp73-OE MM cells, which promoted the immune evasion of MM cells. CD47 emits a “do not eat me” signal in healthy cells and thus prevents cell phagocytosis by macrophages; however, its expression is downregulated on old and redundant cells, promoting cell elimination from macrophages. CD47 on the MM cell surface is overexpressed by the DNp73/MYC pathway to help prevent MM cells from being killed by innate immune cells. PD-1 and PD-L1 inhibitors have been increasingly utility in cancer therapy. Anti-PD-1/PD-L1 antibody-based inhibitors induce durable tumor remission in patients with diverse advanced cancers, and thus, the expression of PD-L1 on tumor cells and other cells in the tumor microenvironment is of major clinical relevance (53). However, the results of a clinical trial in which PD-1/PD-L1 inhibitor monotherapy in MM patient treatment was unsatisfactory (54). Kelly et al. suggested that strategies for increasing PD-L1 levels may potentially prime malignant cells with low PD-L1 expression to become sensitive to anti-PD-1/PD-L1 antibody-based blockade (55). In the study, patients with enhanced DNp73 expression exhibited PD-L1 overexpression, suggesting that DNp73 functions as a biomarker for predicting the efficacy of PD-L1 inhibitors in MM. Targeting DNp73 may be a promising new strategy for treating MM.

## Data Availability

The datasets presented in this study can be found in online repositories. The names of the repository/repositories and accession number(s) can be found below: https://bigd.big.ac.cn/gsa-human/browse/HRA004574, HRA004574.
