# Supplementary material for: DNp73 enhances tumor progression and immune evasion in multiple myeloma by targeting the MYC and MYCN pathways
Source: Front Immunol. 2024 Sep 24;15:1470328. doi: 10.3389/fimmu.2024.1470328 (PMC11459316; doi:10.3389/fimmu.2024.1470328)
Supplement: Supplementary file 1 [file DataSheet1.docx]

**DNp73 enhances tumor progression and immune evasion in multiple myeloma by targeting the MYC and MYCN pathways**

**Supplemental figures**

**Supplemental tables**

**Supplemental Figures**

**Supplementary Figure 1. DNp73 is upregulated via the miRNA-15a/NF-κB pathway in MM cells**

**A.** The map of p65 binding to the DNp73 promoter was obtained from the UCSC genome browser ChIP-seq data.

**B.** Schematic diagram of the binding site for p65 (RelA) and the promoter region of DNp73 located 2 kb upstream of the translation start site (TSS).

**C.** Quantification of western blotting in **Figure 1C**.

**D.** RT‒qPCR analysis revealed the expression of miR-15a and DNp73 in CD138^+^ primary MM cells from NDMM patients. The scatter plot shows that DNp73 expression was negatively correlated with miR-15a expression in 10 samples collected from patients with NDMM (r = -0.67, P = 0.03).

**E.** Immunoblotting detection of DNp73 and TAp73 expression in MM cell lines.

**F.** RT‒qPCR of DNp73 and TAp73 expression in MM cell lines.

**Supplementary Figure 2. Enhanced DNp73 expression promotes MM cell growth and drug resistance.**

**A.**Flow cytometric analysis of the cell cycle in ARP1 and OCI-MY5 EV or DNp73-OE cells.

**B.** MM cells were exposed to 2 Gy radiation, and the DNA damage response was detected by immunofluorescence staining with γH2AX foci and analyzed using confocal microscopy. Graphic presentation of the relative γH2AX+ EV and DNp73-OE ARP1 cells. The cells containing > 5 γH2AX foci were recorded as γH2AX+ cells, whose percentage was averaged from at least 100 cells. The number of γH2AX foci without irradiation was normalized to 1. Scale bars, 14 µm.

**C.** The percentage of surviving cells was detected by trypan blue exclusion assay after exposure to 2 Gy radiation.

**D.** Cell apoptosis was evaluated in OCI-MY5 EV and DNp73-OE cells treated with DMSO and epirubicin, followed by Annexin V staining and flow cytometry.

**E.** Apoptosis of ARP1 EV and DNp73-OE cells was evaluated by flow cytometry after treatment with DMSO and melphalan or bendamustine for 24 hours.

**F.** A colony formation assay was conducted with equal numbers of MM cells seeded in semisolid methylcellulose complete medium with or without EPI (100 nM) .

**G.** Tumors from the ARP1 EV and DNp73-OE group mice were collected and photographed. The statistical analysis of tumor weights among different groups for the experimental endpoint.

**H.** The tumor sizes during growth in each group were recorded.

**Supplementary Figure 3. Knockdown of DNp73 inhibits the myeloma cell cycle, metastasis, and DNA damage repair.**

**A.**Flow cytometric analysis of the cell cycle in ARP1 and RPMI 8226/Dox40 Scramble or sh-DNp73 cells.

**B.** Cell proliferation was measured by CCK-8 assay in DNp73 knockdown MM cells.

**C.** Whole-cell lysates of MM cells were subjected to immunoblot analysis with the indicated antibodies.

**D.** The migration and invasion abilities of scramble and shRNA-DNp73 cells were measured by Transwell assay.

**E.** Drug sensitivity was determined by measuring cell apoptosis in ARP1 and RPMI 8226/Dox40 cells expressing shRNA-DNp73 and controls with or without treatment with epirubicin, pomalidomide, or carfilzomib; this was detected by Annexin V staining flow cytometry.

**F.** Knockout (KO) of DNp73 in ARP1 cells using the CRISPR-Cas9 system, and assessment of knockout efficiency by Western blot analysis.

**G.**Whole-cell lysates of MM cells were subjected to immunoblot analysis with the indicated antibodies.

**H.** Response to radiation or carfilzomib in RPMI 8226/Dox40 cells. γH2AX foci were counted 8h after irradiation. After 24 hours of treatment with carfilzomib, count the γH2AX foci. Cells were immunostained with anti-γH2AX (green) and DAPI (blue) and analyzed using confocal microscopy. Graphic presentation of the relative γH2AX+ control and DNp73-KO cells. The cells containing > 5 γH2AX foci were recorded as γH2AX+ cells, whose percentage was averaged from at least 100 cells. The number of γH2AX foci without irradiation was normalized to 1. Scale bars, 14 µm.

**I.** The RPMI 8226/Dox40 cells were exposed to radiation or carfilzomib and the percentage of surviving cells were assayed by trypan blue exclusion.

**Supplementary Figure 4. Knockdown of DNp73 inhibits myeloma cell growth and sensitizes cells to chemotherapeutic drugs.**

**A.** Tumor volume measurements in each group of mice after tumor cell inoculation.

**B.** The statistical analysis of tumor weights among different groups for the experimental endpoint.

**Supplementary Figure 5. RNA-seq indicates that DNp73 activates the MYC-related pathway**

**A.** Heat map showing the expression values of differentially expressed genes between the EV and DNp73 OE groups, scramble and shRNA-DNp73 groups.

**B.** GSEA showed that higher DNp73 expression was positively correlated with DNA repair related genes, and negatively correlated with p53 related genes in ARP1 cells.

**C.** RT‒qPCR analysis of DNp73 and MYCN expression in MM cell lines.

**D.** Quantification of western blotting in **figure 5E**.

**E.** The expression of DNp73, MYC, and MYCN in relapsed refractory MM patients was detected by immunoblotting.

**F**. The correlation analysis between DNp73 and MYC or MYCN protein expression in **Figure 5F** and **Supplementary Figure 4E**.

**G.** Detection of DNp73, MYC and MYCN expression in MM cells using Immunoblotting.

**Supplementary Figure 6. Combination analysis of ChIP-seq and RNA-seq identified that DNp73 acts as a transcription factor to regulate MYCN expression**

**A.** Venn diagram showing overlapping genes between RNA-seq (p<0.01, log2FoldChange>2) and CHIP-seq (The binding site is located within 1000bp upstream and downstream of the TSS).

**B.** The motifs most frequently bound by the DNp73 transcription factor were identified by ChIP-seq.

**Supplementary Figure 7. Anti-CD47 treatment with IMM01 overcomes DNp73-induced immune evasion in MM**

**A.** Kaplan‒Meier analysis was performed in MM patients with high levels of CD47 based on the GEO data of individuals with relapsed MM (GSE9782).

**B.** The protein level of CD47 on myeloma cells was examined through flow cytometric analysis.

**C.** Cells were immunostained with anti-CD47 (orange) and DAPI (blue) and analyzed using confocal microscopy. Scale bars, 14 µm.

**D & E.** Phagocytosis assay. Green signals indicate macrophages engulfing CFSE-labeled myeloma cells, and red signals represent macrophages. Quantitation of the phagocytic index of myeloma cell lines as described in Materials and Methods. Scale bars, 100 µm.

**F.** Xenograft MM mouse model.

**G.** Serum human Ig-Kappa (secreted by ARP1 cells) was detected in mice at the time of killing to indicate tumor burden.

**H.** The expression of miR-15a in ARP1 EV and ARP1 DNp73-OE cells was detected by RT‒qPCR.

The data are displayed as the mean plus SD of three counts, and statistical significance was calculated and represented as the P value. *P < .05, **P < .01, ***P < .001, ****P < .0001.

**Supplementary Figure 8. Schematic overview of this work.**

**Suppl. Fig.1**

**
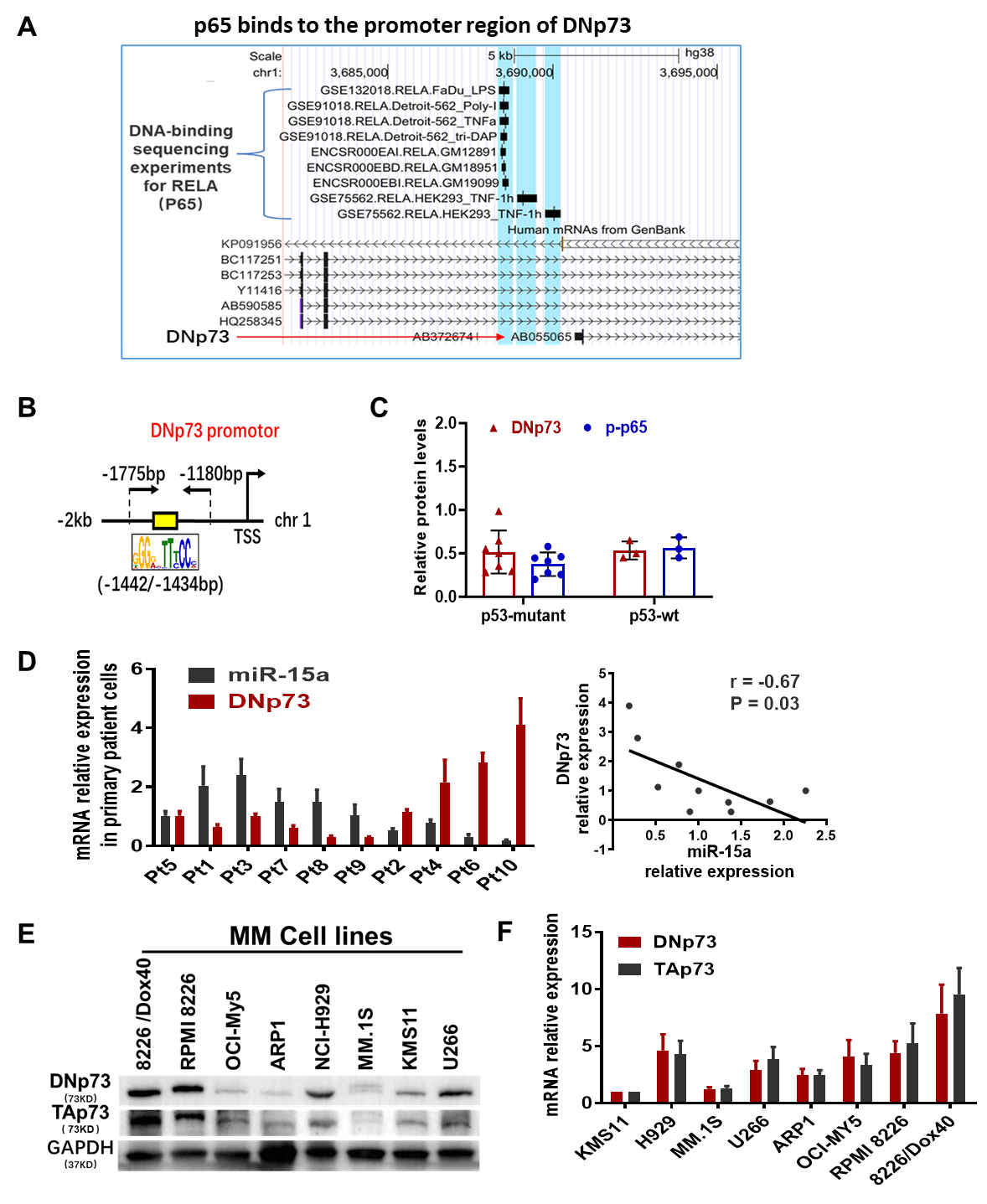
**

**Suppl. Fig.2**

**
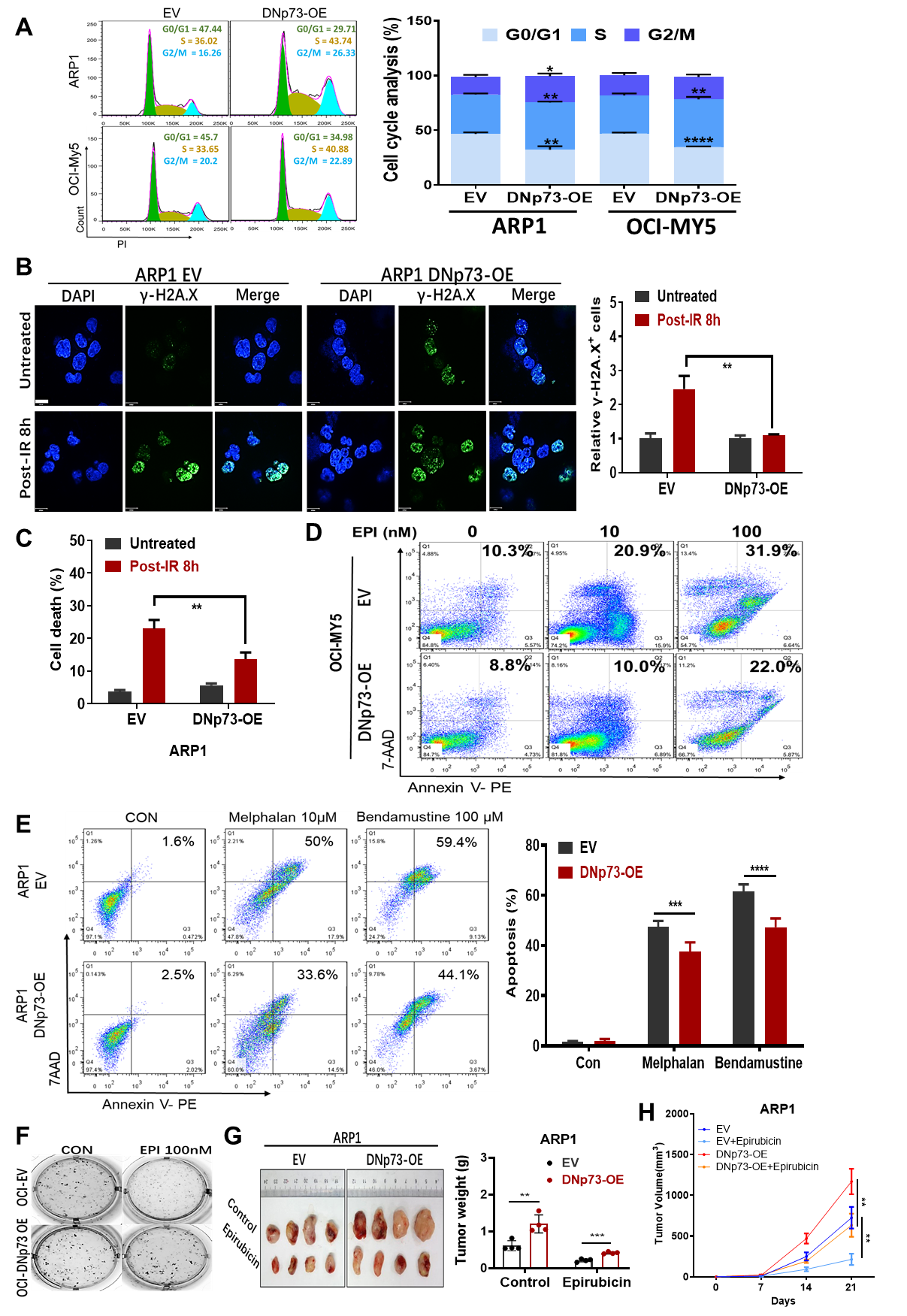
**

**Suppl. Fig.3**

**
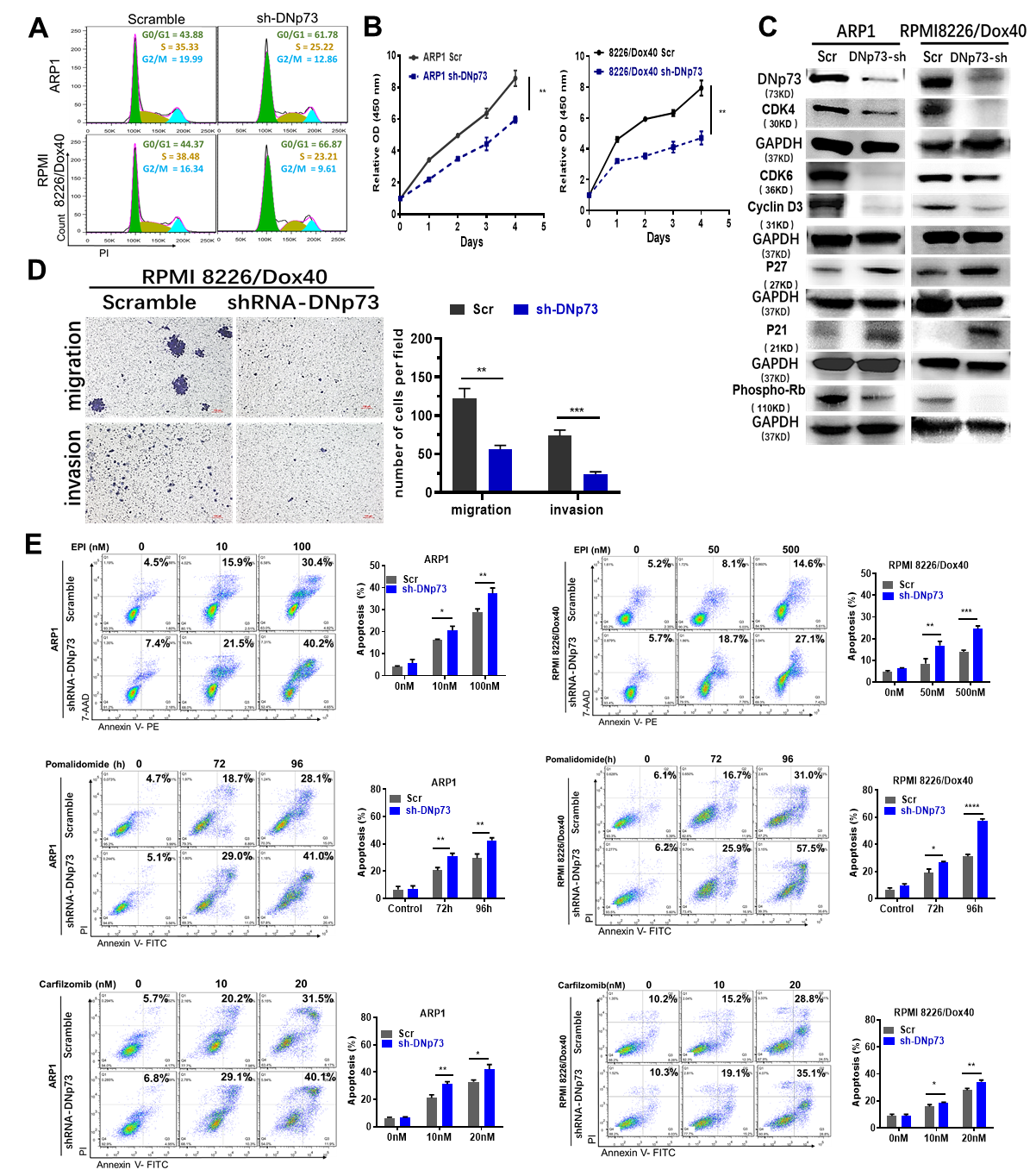
**

**
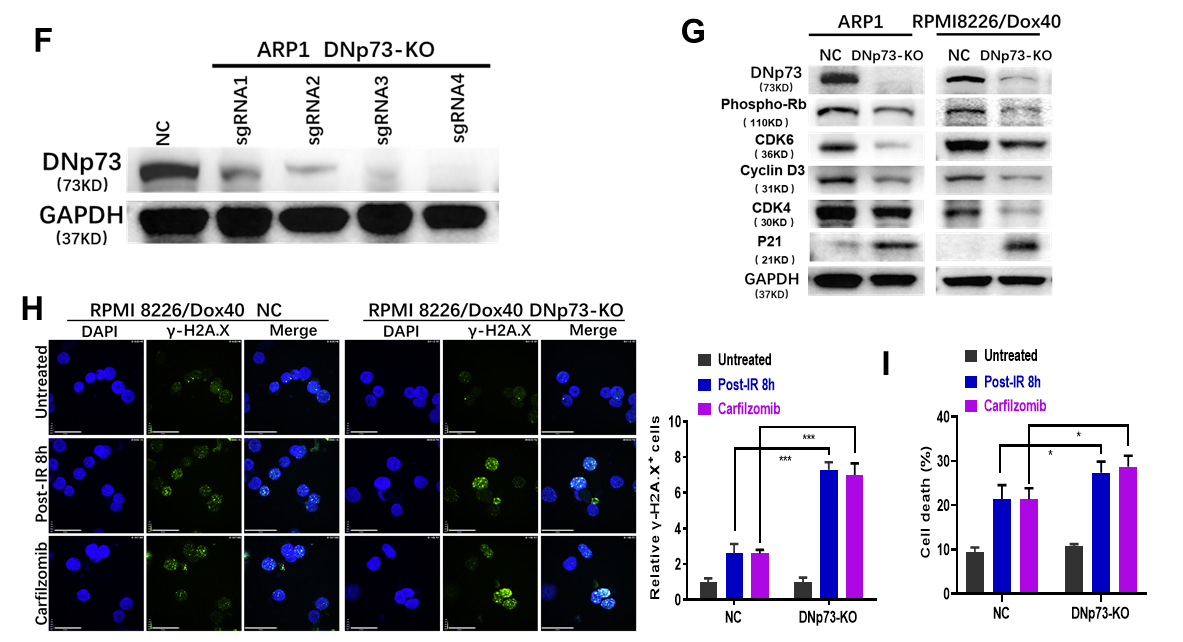
**

**Suppl. Fig. 4**

**
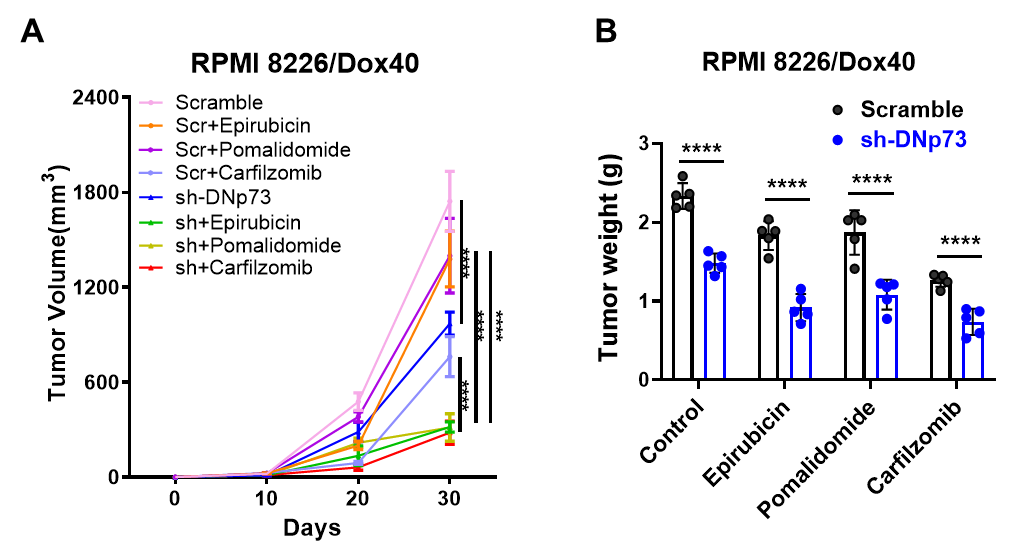
**

**Suppl. Fig. 5**

**
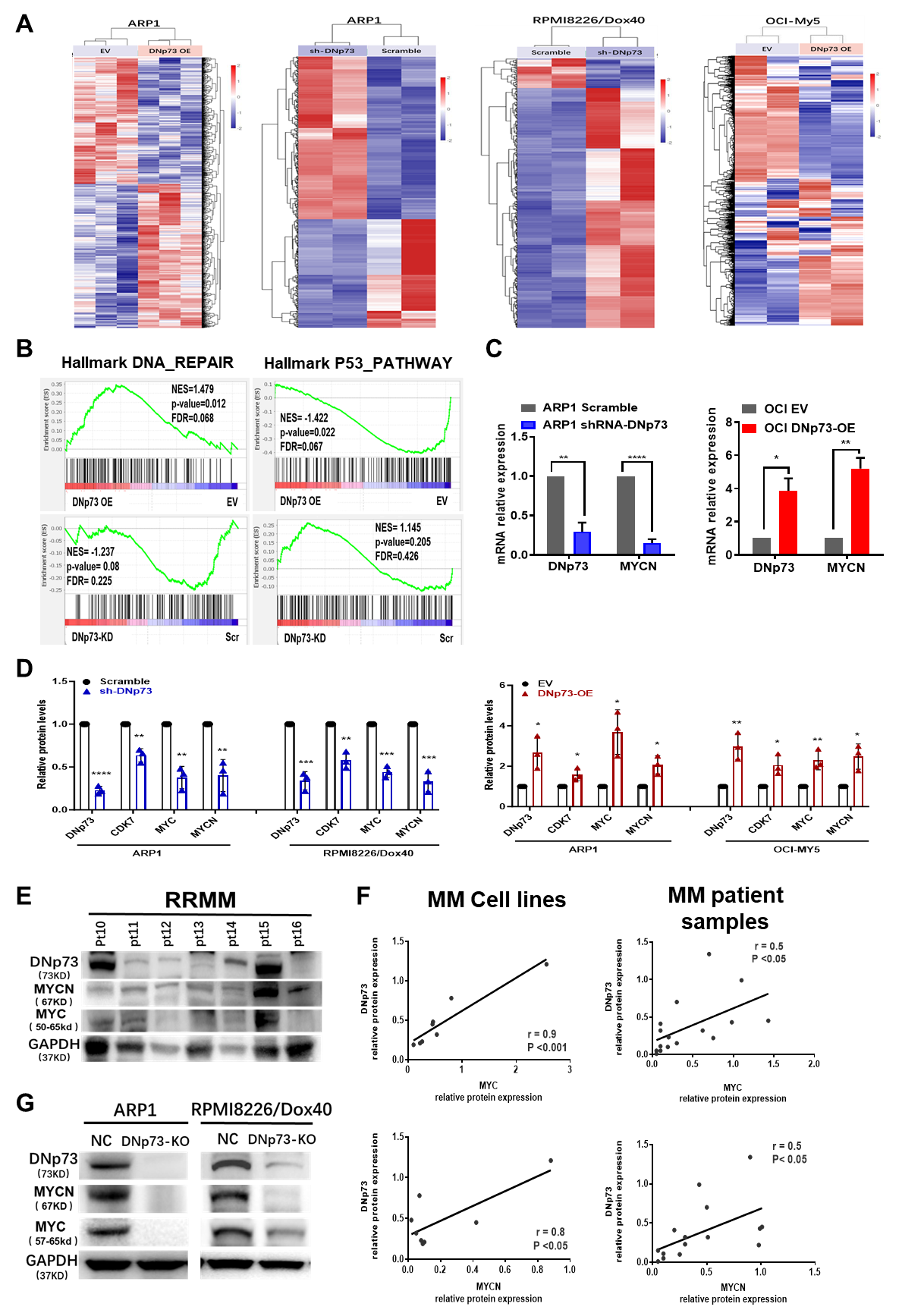
**

**Suppl. Fig. 6**

**
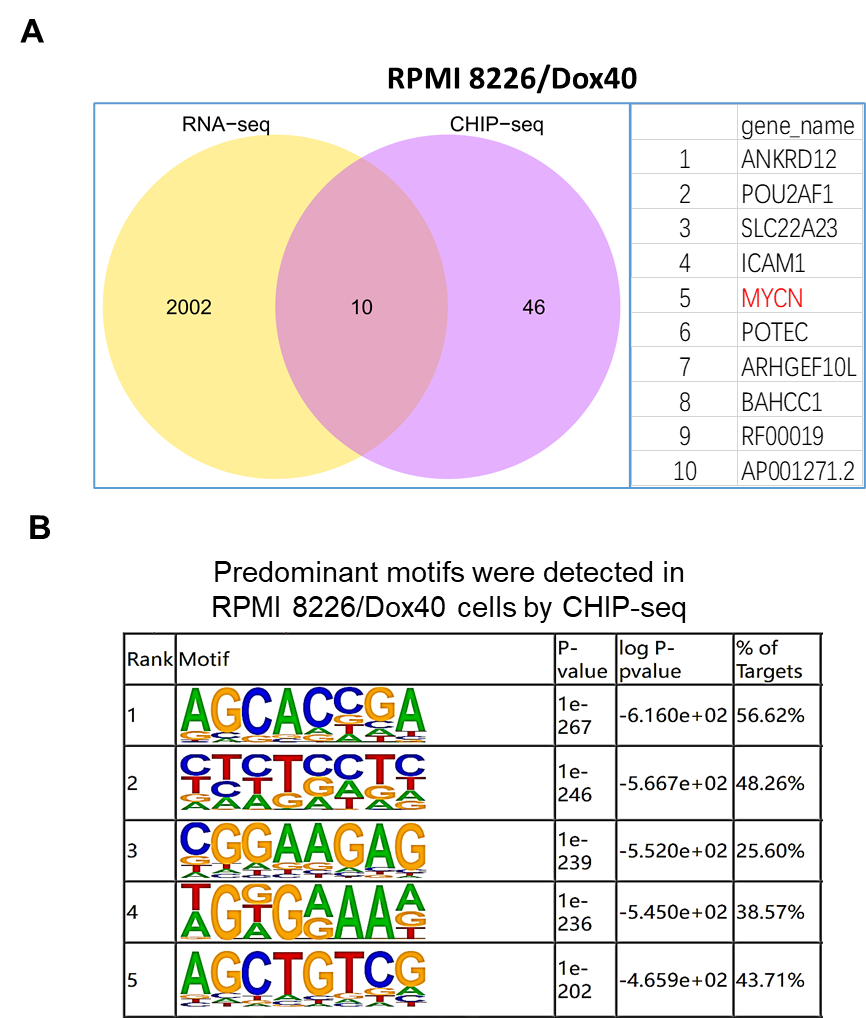
**

**Suppl. Fig.7**

**
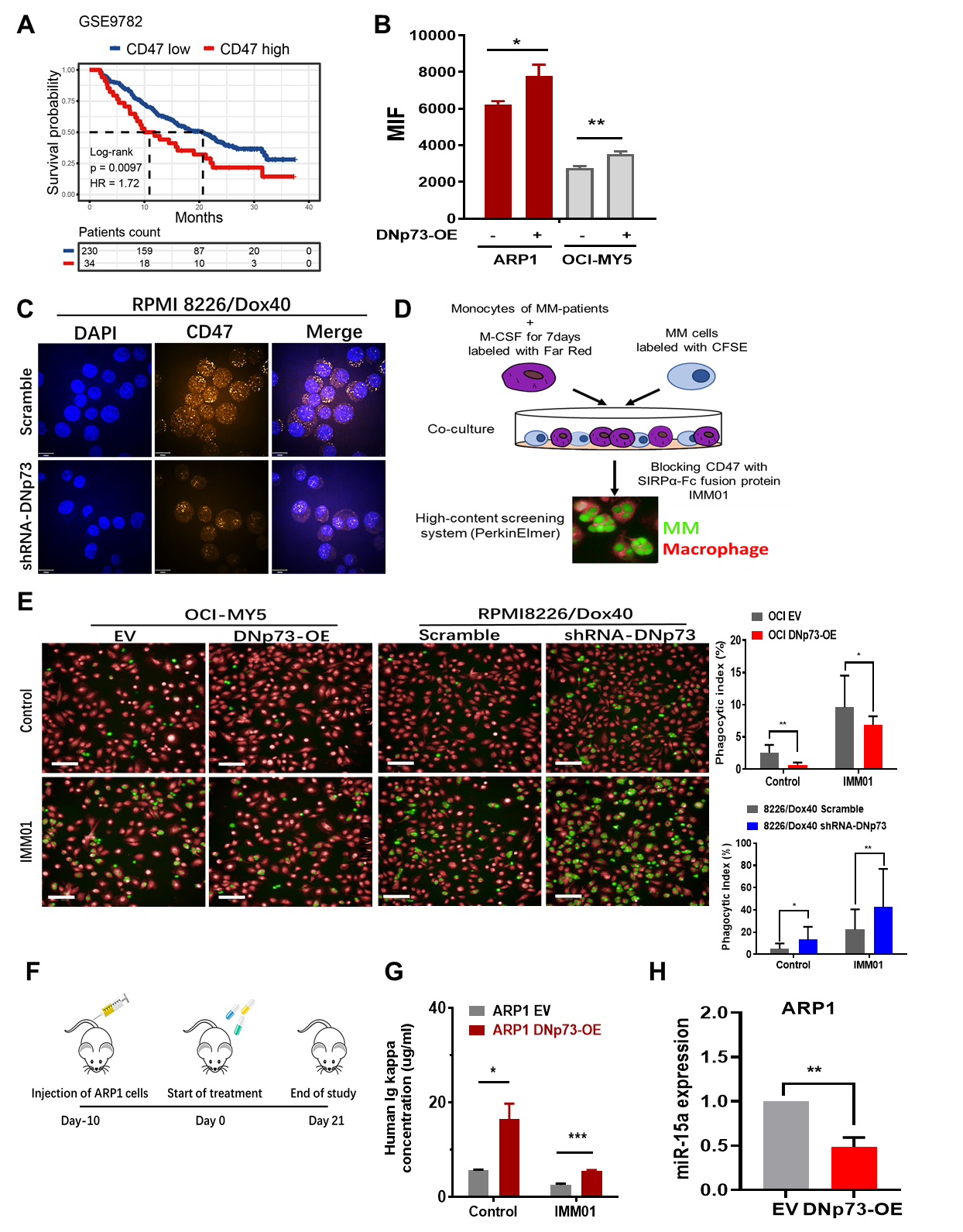
**

**Suppl. Fig. 8**

**
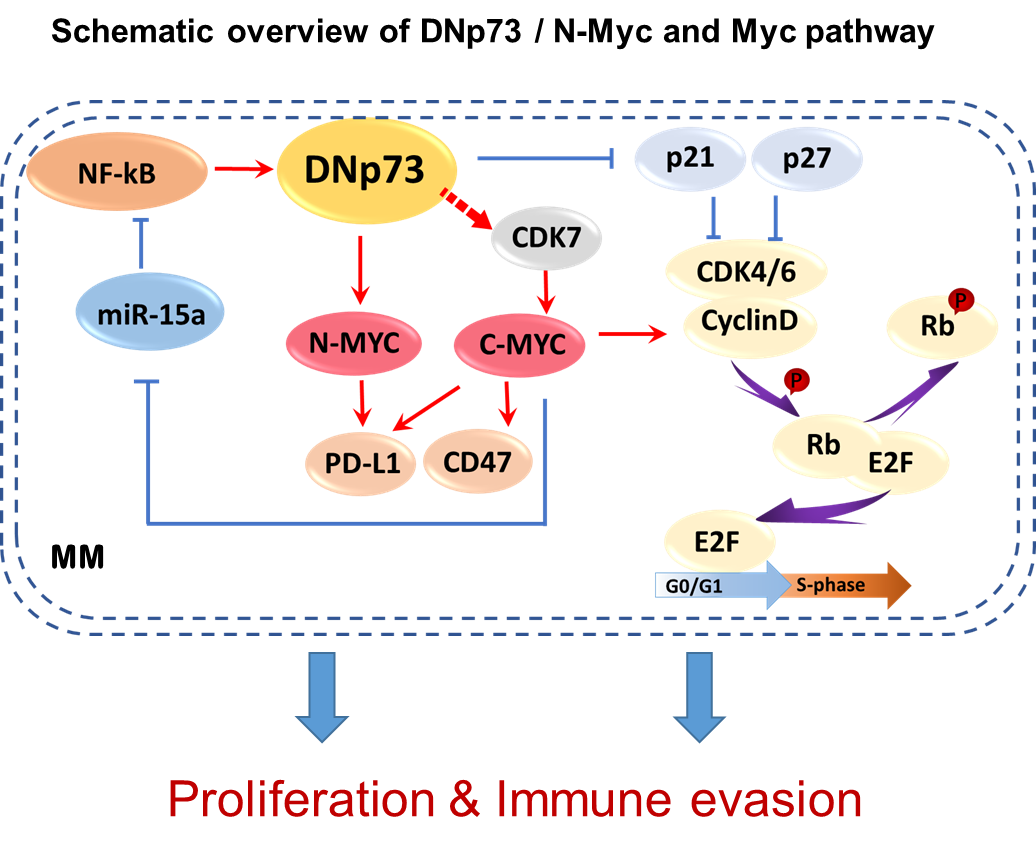
**

**Supplementary Table 1.**  Baseline characteristics of 15 NDMM and 7 RRMM patients

| Characteristics | NDMM patients | RRMM patients |
| --- | --- | --- |
| No. of patients | 15 | 7 |
| Sex |  |  |
| Male | 8 (53%) | 4 (57%) |
| Female | 7 (47%) | 3 (43%) |
| Age/median; range | 56 (33-68) | 55 (36-68) |
| BM-infiltration (%) median; range | 60 (5-90) | 55 (5-85) |
| Aberrant Cytogenetics |  |  |
| 1q21 gain | 7 (47%) | 3 (43%) |
| 13q14 deletion | 8 (53%) | 4 (57%) |
| IgH translocation | 5 (33%) | 3 (43%) |
| 17p deletion | 2 (13%) | 1 (7%) |
| MYC rearrangement | 1 (7%) | 1 (7%) |
| ISS stage |  |  |
| Ⅰ | 1 (7%) | 0 |
| Ⅱ | 6 (40%) | 3 (43%) |
| Ⅲ | 8 (53%) | 4 (57%) |
| RISS stage |  |  |
| Ⅰ | 0 | 0 |
| Ⅱ | 8 (53%) | 3 (43%) |
| Ⅲ | 7 (47%) | 4 (57%) |
| Hemoglobin (g/L) median; range | 85 (64-124) | 87 (59-110) |

ISS: International Staging system

RISS: Revised International Staging System

**Supplementary Table 2.** Antibodies

| **Antibodies** | **Sources** | **Catlog #** | **Applications** |
| --- | --- | --- | --- |
| NF-κB p65 | Cell signaling technology | 8242 | ChIP (1:100), WB (1:1000) |
| phospho-NF-κB p65(Ser536) | Cell signaling technology | 3033 | WB (1:1000) |
| IκBα | Cell signaling technology | 4812 | WB (1:1000) |
| Phospho-IκBα (Ser32) | Cell signaling technology | 2859 | WB (1:1000) |
| c-Myc | Cell signaling technology | 5605 | WB (1:1000) |
| p21 | Cell signaling technology | 2947 | WB (1:1000) |
| p27 | Cell signaling technology | 3688 | WB (1:1000) |
| CDK4 | Cell signaling technology | 12790 | WB (1:1000) |
| CDK6 | Cell signaling technology | 13331 | WB (1:1000) |
| CDK7 | Cell signaling technology | 2090 | WB (1:1000) |
| Phospho-Rb (Ser608) | Cell signaling technology | 8147 | WB (1:1000) |
| PD-L1 | Cell signaling technology | 60475 | WB (1:1000) |
| GAPDH | Cell signaling technology | 5174 | WB (1:1000) |
| N-Myc | Santa Cruz Biotechnology | sc-53993 | WB (1:1000) |
| Delta Np73 | Novus | NBP2-24873 | ChIP (1:20), WB (1:1000) |
| p73 | Novus | NBP2-24737 | WB (1:1000) |
| Cyclin D3/CCND3 | abcam | ab112034 | WB (1:1000) |
| rabbit IgG-HRP | Cell signaling technology | 7074 | WB (2nd Ab) (1:2000) |
| mouse IgG-HRP | Cell signaling technology | 7072 | WB (2nd Ab) (1:2000) |

WB, western blot. ChIP, chromatin immunoprecipitation. HRP, horseradish peroxidase.
